# S-nitrosylation-mediated activation of a histidine kinase represses the type 3 secretion system and promotes virulence of an enteric pathogen

**DOI:** 10.1038/s41467-020-19506-1

**Published:** 2020-11-13

**Authors:** Dan Gu, Yibei Zhang, Qiyao Wang, Xiaohui Zhou

**Affiliations:** 1 Department of Pathobiology and Veterinary Science, University of Connecticut, Storrs, 06269 CT USA; 2 College of Bioscience and Biotechnology, Yangzhou University, Yangzhou, China; 3 State Key Laboratory of Bioreactor Engineering, East China University of Science and Technology, 200237 Shanghai, China

**Keywords:** Kinases, Bacterial pathogenesis, Pathogens, Nitrosylation

## Abstract

*Vibrio parahaemolyticus* is the leading cause of seafood-borne diarrheal diseases. Experimental overproduction of a type 3 secretion system (T3SS1) in this pathogen leads to decreased intestinal colonization, which suggests that T3SS1 repression is required for maximal virulence. However, the mechanisms by which T3SS1 is repressed in vivo are unclear. Here, we show that host-derived nitrite modifies the activity of a bacterial histidine kinase and mediates T3SS1 repression. More specifically, nitrite activates histidine kinase sensor VbrK through *S*-nitrosylation on cysteine 86, which results in downregulation of the entire T3SS1 operon through repression of its positive regulator *exsC*. Replacement of cysteine 86 with a serine (VbrK C86S mutant) leads to increased expression of inflammatory cytokines in infected Caco-2 cells. In an infant rabbit model of infection, the VbrK C86S mutant induces a stronger inflammatory response at the early stage of infection, and displays reduced intestinal colonization and virulence at the later stage of infection, in comparison with the parent strain. Our results indicate that the pathogen *V. parahaemolyticus* perceives nitrite as a host-derived signal and responds by downregulating a proinflammatory factor (T3SS1), thus enhancing intestinal colonization and virulence.

## Abstract (web summary)

*Vibrio parahaemolyticus* causes seafood-borne diarrheal diseases. Here, the authors show that the pathogen uses a histidine kinase to sense host-derived nitrite and downregulate a proinflammatory type 3 secretion system, thus enhancing intestinal colonization and virulence.

## Introduction

The type 3 secretion system (T3SS) is an important virulence determinant employed by a variety of gram-negative bacterial pathogens to cause diseases in humans. The primary function of the T3SS is to translocate effector proteins into the host cells^1^, where they hijack host signaling pathways for the benefit of infection^2,3^. For enteric pathogens, the expression of the T3SS is tightly controlled in response to environmental and host cues. For example, expression of the T3SS encoded in the *Yersinia* pYV plasmid is repressed at 30 °C and is activated at 37 °C. This thermal regulation of T3SS is achieved by the repressor protein YmoA, which is degraded at 37 °C and thus unable to repress T3SS expression^4^. *Salmonella* SPI1 T3SS is induced by low oxygen tension and high osmolarity conditions^5,6^, whereas SPI2 is activated by acidic pH or a low concentration of Mg^2+^,^7^. Tight control of T3SS genes in pathogenic bacteria ensures maximal fitness during different stages and at different sites of the infection^1^.

*Vibrio parahaemolyticus* is a gram-negative bacterium that causes seafood-borne gastroenteritis worldwide^8^. The thermostable direct hemolysin (TDH) is the main exogenous toxins of *V. parahaemolyticus* that form pores on the host cell membranes in vitro^9^ and is partially involved in enterotoxicity in rabbit ileal loop model^10^, but studies using an infant rabbit model showed that TDH does not play significant roles in pathogenesis in vivo^11^. Two type 3 secretion systems (T3SS1 and T3SS2) have been identified in the genome of *V. parahaemolyticus*^12^. Genetic, biochemical, and structural studies demonstrated that T3SS2 gene expression is activated by bile salt through its direct binding to the transmembrane protein VtrC^13,14^. In vitro, T3SS2 has been shown to contribute to bacterial internalization into the non-phagocytic cells^15,16^ and such process is dependent on its effector VopC’s capacity to deamidate Rac1 and CDC42 and formation of actin stress fibers^17^. Studies using rabbit ileal loop model revealed that T3SS2 is important for enterotoxicity and fluid accumulation^18^. In the infant rabbit infection model, T3SS2 plays an essential role in intestinal colonization and diarrheal disease^11^ and these two processes can be genetically separated by an internal fragment of the effector VopZ^19^. In a recently developed germfree mouse infection model, T3SS2 was shown to contribute to bacterial invasion into the intestinal mucosa and pathology^20^. Deletion of the effector VopC resulted in significant reduction in bacterial mucosal invasion and gastroenteritis, but not pathology in this germfree mouse model^20^. Together, these results highlight the importance of T3SS2 in the pathogenesis of *V. parahaemolyticus* in vivo.

T3SS1 expression is genetically regulated by the ExsACDE cascade in which the master transcription factor ExsA positively regulates T3SS1 expression, while ExsD negatively regulates T3SS1 expression^21^. ExsC is involved in the positive regulation of T3SS1 by binding to ExsD^22^, while ExsE is involved in the negative regulation of T3SS1 potentially by disrupting the interaction between ExsC and ExsD^23^. In vitro, T3SS1 is responsible for orchestrated host cell death that includes VopQ-dependent autophagy, cell rounding and cell lysis^24–26^. Although the genetic pathway that regulates T3SS1 expression has been elucidated, the environmental signals and the sensors that respond to these signals are largely unknown. Furthermore, although it is known that T3SS1 is responsible for cytotoxicity, autophagy induction^26^ and stimulation of the inflammatory response in vitro^27,28^, its roles in vivo are largely unknown primarily due to the experimental observations that deletion of T3SS1 does not have significant effect on virulence in infant rabbit^11,19^, ileal rabbit loop^29^ and germfree mouse^20^ infection models. Our previous studies have shown that deletion of the T3SS1 negative regulator ExsD results in decreased intestinal colonization, which indicates that repression of T3SS1 is essential for maximal virulence^30^. These results also highlight the importance of elucidating the sensors and host environmental cues that repress the expression of the T3SS in vivo.

One of the most important mechanisms through which bacterial pathogens sense environmental signals is the two-component system (TCS)^31^. The prototypical TCS is composed of one histidine kinase (HK) and one response regulator (RR). HK is typically located in the inner membrane and senses environmental cues. Once it senses a signal, HK autophosphorylates itself, leading to its activation. Active HK transfers its phosphate group to the cytoplasmic RR, and the active RR subsequently alters gene expression by directly binding to the promoters of its target genes^32,33^. The genome of *V. parahaemolyticus* contains 48 predicted HKs^12^, and we have previously shown that one of these HK, VbrK, can sense β-lactam antibiotics and subsequently activate lactamase gene expression to mediate bacterial resistance to β-lactam antibiotics^34^.

In this work, we show that host-derived nitrite activates VbrK through *S*-nitrosylation and subsequently suppresses T3SS1 expression to dampen the inflammatory response and enhance colonization and virulence in the small intestine. These results not only reveal that nitrite serves as an environmental cue to activate HK and subsequently repress T3SS1 expression but also uncover a molecular mechanism through which HK is activated via *S*-nitrosylation.

## Results

### Identification of the HKs that regulate T3SS1 expression

To test the hypothesis that one of the TCSs is involved in T3SS1 regulation, we constructed 48 mutants in which each predicted HK was individually deleted from the chromosome of *V. parahaemolyticus*. We then determined the secretion of a T3SS1 substrate (Vp1656, VopD1) by these mutants under growth conditions consisting of LB medium supplemented with carbenicillin (except for the *vpa0920* mutant because it is not resistant to β-lactam antibiotics^34^). The secretion of VopD1 was considered to reflect T3SS1 activation because the secretion process generally requires expression of all the genes encoding the T3SS machinery. As expected and consistent with our previous results, VopD1 was not secreted by the WT in LB medium (Supplementary Fig. 1) because LB medium lacks the signals needed to induce the expression of the master transcription factor ExsA for T3SS1^21^. As a positive control, forced overexpression of ExsA by a plasmid (WT:p*exsA*) induced the secretion of VopD1 by the WT (Supplementary Fig. 1). Deletion of *vpa0920* and *vpa1100* induced the secretion of VopD1 into the supernatant (Supplementary Fig. 1, lanes 6 and 9), which indicates that these two HKs play a role in repressing T3SS1 gene expression in LB medium. In contrast, the remaining 46 HK mutations exerted no significant effects on the secretion of VopD1 (Supplementary Fig. 1). Notably, the *vpa0920* mutant was associated with notably more significant secretion of VopD1 than the *vpa1100* mutant (Supplementary Fig. 1, compare lane 6 and 9). Thus, in this study, we focused on characterizing the function of Vpa0920 in sensing environmental cues and regulating T3SS1 gene expression.

### The VbrK/VbrR pair regulates the expression of T3SS1

In our previous work, we showed that Vpa0920/Vpa0919 is a TCS pair (HK/RR) that controls β-lactam resistance in *V. parahaemolyticus*^34^. To further verify that T3SS1 activity is also controlled by VbrK/VbrR and the phosphorylation status of VbrK/VbrR, we determined the secretion of VopD1 by the strains shown in Supplementary Fig. 2. As expected, VopD1 was secreted into the supernatant by both Δ*vbrK* and Δ*vbrR*, whereas its respective complement strains (Δ*vbrK:*p*vbrK*, Δ*vbrR*:p*vbrR*) could not secrete VopD1 (Supplementary Fig. 2a), which indicated that VbrK/VbrR is a TCS pair (HK/RR) that represses T3SS1 activity in *V. parahaemolyticus*. HK and RR rely on its conserved histidine and aspartate residues, respectively, for its phosphorylation and functionality. Thus, we determined whether a mutation in the conserved histidine and aspartate residues in VbrK and VbrR would impair their regulation of T3SS1 expression. We complemented Δ*vbrK* and Δ*vbrR* with p*vbrK*^*H286A*^ (plasmid harboring VbrK with the histidine residue mutated to an alanine at the conserved 286^th^ amino acid) and p*vbrR*^*D51A*^ (plasmid harboring VbrR with the aspartate residue mutated to an alanine at the conserved 51st amino acid), respectively. The results showed that VopD1 could be secreted by these complement strains, albeit at a lower level than that observed with the Δ*vbrK* and Δ*vbrR* mutant strains (Supplementary Fig. 2a), which indicated that these conserved phosphorylation sites are important for the functionality of VbrK and VbrR in repressing T3SS1 expression. The results from the quantitative RT-PCR (qRT-PCR) analysis of *vopD1* expression were consistent with the western blotting results, which indicated that VbrK/VbrR represses T3SS1 function at the transcriptional level (Supplementary Fig. 2b). We further determined the role of VbrK/VbrR in regulating the expression of other T3SS1 genes within the operon. We selected seven genes (including both structural and effector genes) and qRT-PCR analysis showed that all seven genes were upregulated in the Δ*vbrK* and Δ*vbrR* mutants and that the expression levels of these genes in the respective complement strains were restored to the wild-type level (Supplementary Fig. 2c–i). In contrast, the expression of these seven genes in Δ*vbrK:pvbrK*^*H286A*^ and Δ*vbrR:*p*vbrR*^*D51A*^ was comparable to that in Δ*vbrK* and Δ*vbrR*, respectively (Supplementary Fig. 2c–i). These findings demonstrated that VbrK/VbrK regulates the expression of both structural and effector genes of T3SS1 at the transcriptional level.

### VbrK/VbrR regulates T3SS1 through ExsACD

**Fig. 1:**
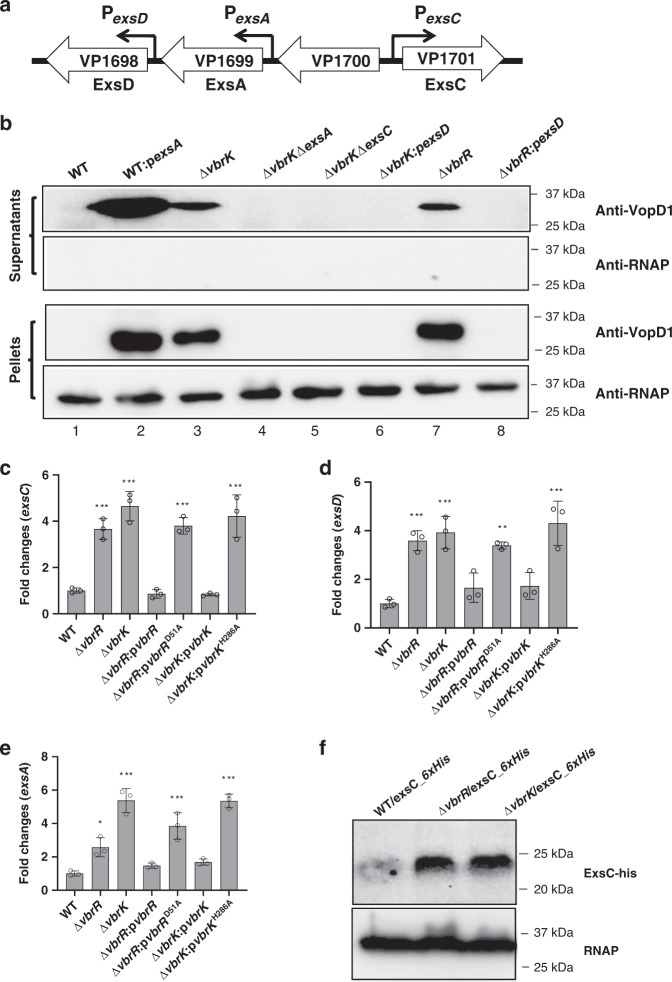
ExsA, ExsC, and ExsD are involved in the VbrK/VbrR-mediated regulation of T3SS1 gene expression in *V. parahaemolyticus*. **a** Schematic representation of *exsA* (*vp1699*), *exsC* (*vp1701*), and *exsD* (*vp1698*) operon as well as their respective promoters (P_*exsA*_, P_*exsC*_, and P_*exsD*_). **b** Western blot analysis of VopD1 (Vp1656) in the supernatant and pellet of indicated *V. parahaemolyticus* strains. RNAP was used to ensure equal amount of protein samples were loaded for the pellet samples. Representative image was shown from three biologically independent experiments. **c**–**e** Quantitative RT-PCR analysis of *exsC* (**d**)*, exsD* (**d**) and *exsA* (**e**) expression in the indicated strains. Bars with standard deviation (*n* = 3 biologically independent samples) indicate average fold changes relative to WT. Statistical significance was calculated using one-way ANOVA with Bonferroni post-hoc analysis. Asterisks indicate *P* values *P < 0.05, **P < 0.005, and ***P < 0.0005 compared to gene expression in WT. **f** Western blot analysis of ExsC expression in WT or *vbrK* and *vbrR* mutant strains. 6xHis tag was inserted in the C-terminus of *exsC* in the genome of WT, *∆vbrK*, and ∆*vbrR* (Lanes 1, 2, 3, respectively). Samples of bacterial pellet from the indicated strains were blotted with anti-His antibody. RNAP was used as loading control.

Previous studies have indicated that the master transcription factor ExsA directly binds to the promoters of T3SS1 genes and positively regulates the expression of T3SS1 genes^21^. ExsD negatively regulates T3SS1 gene expression by binding to ExsA, whereas ExsC positively regulates T3SS1 gene expression by binding to ExsD and thus releasing ExsA^22^. The genes of *exsACD* have their own promoters, namely, P_*exsA*_, P_*exsC*_, and P_*exsD*_ (Fig. 1a). We determined whether ExsA, ExsC, and ExsD are involved in VbrK/VbrR-mediated T3SS1 gene regulation. VopD1 was not expressed in and secreted by the Δ*vbrK*Δ*exsA* or Δ*vbrK*Δ*exsC* double-mutant strains (Fig. 1b, lanes 4 and 5). Furthermore, VopD1 was not expressed in and secreted by Δ*vbrK* and Δ*vbrR* strains overexpressing ExsD (Fig. 1b, lane 6 and 8). These results indicated that *exsA*, *exsC*, and *exsD* are involved in VbrK/VbrR-mediated T3SS1 regulation. We subsequently investigated whether VbrK/VbrR regulates the expression of *exsA, exsC*, and *exsD*. Quantitative RT-PCR analysis showed that *exsA, exsC,* and *exsD* were upregulated in the Δ*vbrK* and Δ*vbrR* strains compared to the WT strain, and the expression of these genes in the complement strains (but not the phosphorylation-deficient complement strains) was lower than that in the Δ*vbrK* and Δ*vbrR* strains (Fig. 1c–e). Furthermore, western blotting showed that the expression of ExsC was upregulated in the Δ*vbrK* and Δ*vbrR* strains compared to that in the WT strain (Fig. 1f). Taken together, these studies indicated that VbrK/VbrR controls the expression of ExsACD.

### VbrR directly binds to the exsC promoter region to repress its transcription

**Fig. 2:**
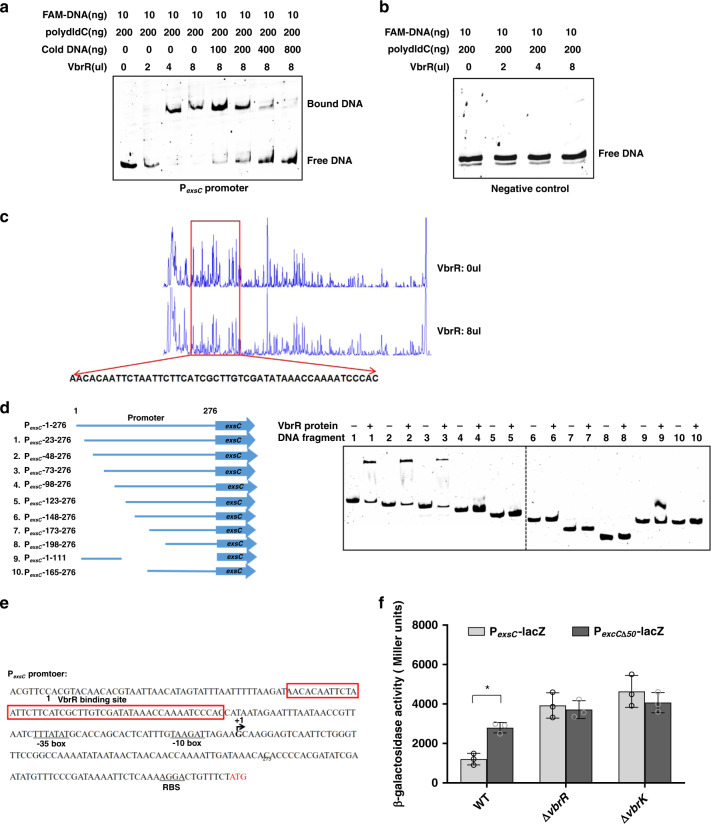
Identification of VbrR-binding site in the promoter of *exsC* and its role in VbrK/VbrR-mediated ExsC repression. **a** EMSA analysis of binding between purified VbrR and promoter of *exsC* (276 bp in length). **b** EMSA analysis of binding between purified VbrR and promoter of *gyrB* (as a negative control). **c** Footprinting analysis of VbrR binding to *exsC* promoter. Electropherograms of a DNase I digest of the P*exsC* promoter probe after incubation with 0 (upper panel) or 400 ng (lower panel) VbrR. The respective nucleotide sequences that were protected by VbrR were indicated under the red boxed electropherograms. **d** EMSA analysis of the binding between VbrR and *exsC* promoter with various truncations. A total of ten PCR fragments for *exsC* promoter (left panel) were analyzed for their binding with VbrR (right panel). **e** Nucleotide sequences (276 bp) of *exsC* promoter (P*exsC*). VbrR-binding site was boxed in red, and −35 box, −10 box and RBS sequences were underlined. Start codon of *exsC* (ATG) was shown in red. **f** Promoter activity of *exsC* in WT, ∆*vbrK*, and ∆*vbrR* strains. All error bars represent mean ± standard deviation (*n* = 3 biologically independent experiments). Statistical significance was calculated using two-tailed multiple *t* test with Bonferroni correction. An asterisk indicates indicate *P* values (*P* = 0.006809).

We showed that VbrK/VbrR regulated the expression of ExsA, ExsC, and ExsD (Fig. 1c–f). However, whether VbrK/VbrR directly or indirectly regulates the expression of these proteins is unclear. We thus determined whether VbrR, the RR that harbors DNA-binding domains, binds to the *exsA*, *exsC,* or *exsD* promoters. Electrophoretic mobility shift assays (EMSAs) showed that mixing the purified VbrR protein with the FAM-labeled *exsC* promoter (276 bp upstream of the *exsC* start codon) (Fig. 2d, e) resulted in a shifted band (Fig. 2a). The addition of nonlabelled cold DNA changed the shifted bands to an unshifted position (Fig. 2a), which indicated that VbrR specifically binds to the *exsC* promoter. VbrR did not bind to the negative control promoter of *gyrB* (Fig. 2b) and to the *exsA* and *exsD* promoters or *vp1677* promoter (Supplementary Fig. 3–c), which suggested that VbrR represses ExsC expression by directly binding to its promoter. In contrast, VbrR represses the transcription of *exsA* and *exsD* indirectly, possibly due to reduced expression of *exsC*. To determine the binding sites of VbrR at the *exsC* promoter, a dye-primer-based DNase I footprinting assay was performed using both strands of the *exsC* promoter. We compared the electropherograms with (Fig. 2c, lower panel) and without VbrR (Fig. 2c, upper panel), and the results showed specific VbrR-protected regions (50 bp from nucleotides position 48 to position 97) in the *exsC* promoter region (Fig. 2c, e, sequences in the red boxes). To confirm the DNase I footprinting results, we amplified DNA fragments with different truncations in the *exsC* promoter, as shown in Fig. 2d (left panel). EMSA results indicated that VbrR bound DNA fragments 1, 2, 3, and 9 but not DNA fragments 4–8 and 10 (Fig. 2d, right panel). These results indicated that the *exsC* promoter region spanning from the nucleotides position 73–97 is responsible for its binding with VbrR (Fig. 2d), which is consistent with the footprinting results that nucleotide position 48–97 of the *exsC* promoter was protected by VbrR (Fig. 2c). Given this direct binding, we subsequently determined whether this specific binding region is required for the VbrR-mediated regulation of *exsC*. We constructed transcriptional fusions between P_*exsC*_ and lacZ (P_*exsC*_-lacZ) and between P_*exsC*Δ50_ and lacZ (P_*exsC*Δ50_-lacZ) in which the 50-bp VbrR-binding region (48–97) was deleted from the *exsC* promoter, and compared the transcriptional activities of these two fusions in the WT or VbrK/VbrR mutants. The promoter activity of P_*exsC*_-lacZ in the Δ*vbrK* and Δ*vbrR* mutants was higher than that in the WT, which indicated that VbrK/VbrR represses the expression of *exsC* (Fig. 2f). Furthermore, the promoter activity of P_*exsC*Δ50_-lacZ was significantly higher than that of P_*exsC*_-lacZ in the WT (Fig. 2f), which indicated that the VbrR-binding site is required for VbrK/VbrR-mediated *exsC* repression. These results revealed that VbrR directly binds to the promoter of *exsC* to repress the expression of *exsC* in *V. parahaemolyticus*.

### The sigma factor Vp2210 is involved in VbrK/VbrR-mediated T3SS1 repression

**Fig. 3:**
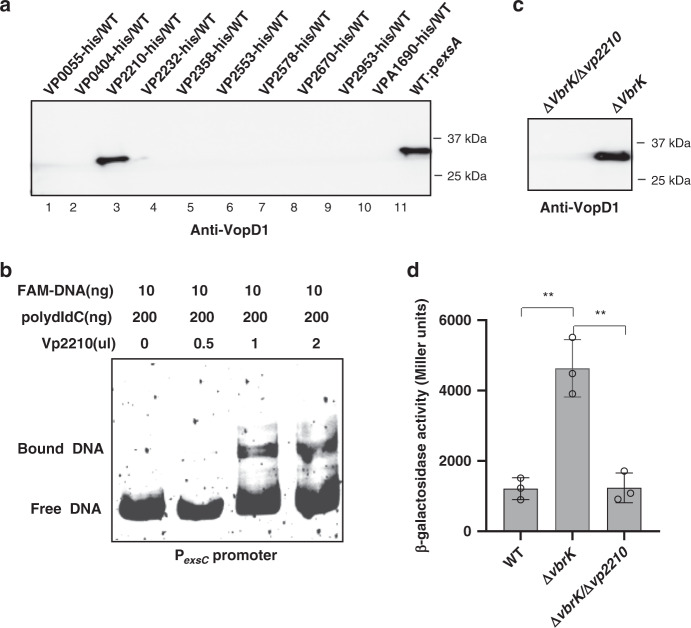
Predicted sigma factor Vp2210 is involved in VbrK-mediated *exsC* repression. **a** Western blot analysis of VopD1 in the supernatant of WT overexpressing individual predicted sigma factor. **b** EMSA analysis of binding between purified protein Vp2210 and the *exsC* promoter. **c** Western blot analysis of VopD1 in the supernatant of Δ*vbrK* and Δ*vbrK*/Δ*vp2210*. **d** Promoter activity of *exsC* in WT, ∆*vbrK*, and ∆*vbrK*∆*vp2210* strains. All error bars represent mean ± standard deviation (*n* = 3 biologically independent experiments). Statistical significance was calculated using one-way ANOVA with Bonferroni correction. Asterisks indicate *P* values (*P* = 0.0009).

We subsequently determined how the binding of VbrR to the promoter of *exsC* represses its transcription. Sigma factors play essential roles in the modulation of gene expression in response to environmental and physiological cues^35^. We hypothesized that VbrR competes the binding of a sigma factor to the *exsC* promoter and thereby affects *exsC* expression. If this hypothesis is true, the overexpression of this sigma factor would alleviate VbrR-mediated *exsC* repression and thus allow T3SS1 gene expression. Ten predicted sigma factors are encoded in the *V. parahaemolyticus* genome. We overexpressed these sigma factors individually in WT *V. parahaemolyticus* and determined the secretion of VopD1. The results showed that overexpression of the sigma factor Vp2210 (belonging to the sigma 70 family, which contains two conserved regions: region 2.0 and region 4.0) resulted in the secretion of VopD1 by WT *V. parahaemolyticus* (Fig. 3a, lane 3). Overexpression of other nine sigma factors had no effect on VopD1 secretion (Fig. 3a). We further purified Vp2210 and performed EMSA to determine whether the Vp2210 protein can directly bind to the promoter of *exsC*. The results showed that Vp2210 directly binds to the *exsC* promoter region (Fig. 3b) but not the negative control *gyrB* promoter (Supplementary Fig. 3d), which indicated that the binding of Vp2210 to the *exsC* promoter is specific. Deletion of Vp2210 from Δ*vbrR* restored the repression status of *exsC* promoter and T3SS1 (Fig. 3c, d). These results suggested that Vp2210 is required for the increased expression of *exsC* in VbrK mutant.

### Nitrite triggers VbrK phosphorylation and reduces T3SS1 expression

**Fig. 4:**
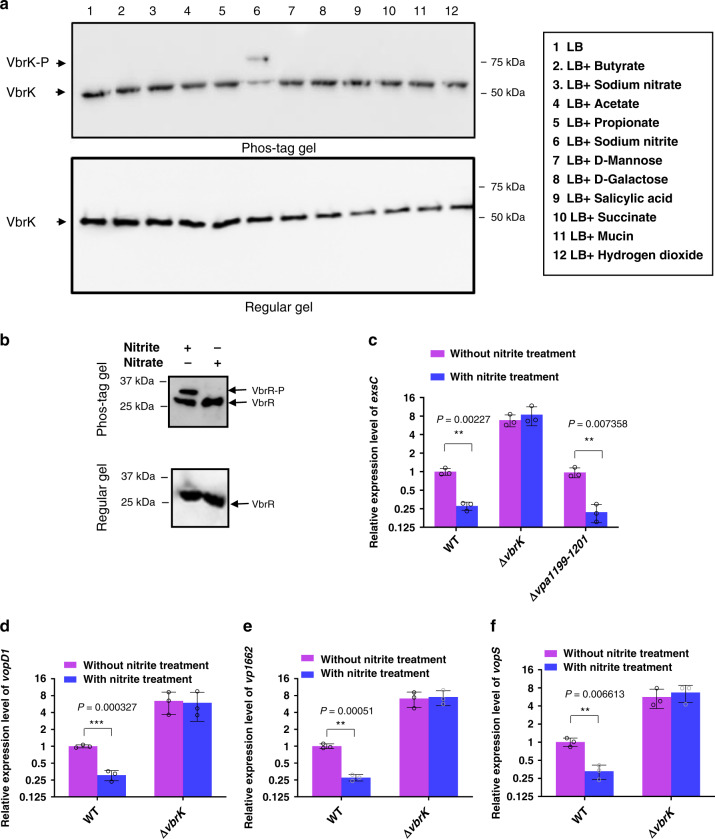
Nitrite activates VbrK and inhibits *exsC* and T3SS1 gene expression. **a** Phosphorylation analysis of VbrK in Δ*vbrK:*p*vbrK_6xHis* cultured in the presence of indicated chemicals (shown on the right panel). Protein samples were separated in Phos-tag gel (upper panel) and regular gel (lower panel) and blotted with anti-His antibody. **b** Analysis of VbrR phosphorylation in Δ*vbrR:*p*vbrR_6xHis* cultured in the presence of nitrite or nitrate (500 μM). Protein samples were separated in Phos-tag gel (upper panel) and regular gel (lower panel) and blotted with anti-His antibody. **c** Quantitative RT-PCR analysis of *exsC* in the indicated strains. Bars represent relative expression of *exsC* compared to that in WT in the absence of nitrite. All error bars represent mean ± standard deviation (*n* = 3 biologically independent experiments). Quantitative RT-PCR analysis of *vopD1* (**d**)*, vp1662* (**e**), and *vopS (vp1686)* (**f**) in the indicated strains. Bars represent relative expression of *vopD1, vp1662*, and *vopS* compared to the respective genes in WT in the absence of nitrite. All error bars represent mean ± standard deviation (*n* = 3 biologically independent experiments). **c**–**f** Statistical significance was calculated using two-tailed multiple *t* test with Bonferroni correction. Asterisks indicate *P* values *P < 0.05, **P < 0.005, and ***P < 0.0005 (exact *P* value for each comparison was shown in the figures).

**Fig. 5:**
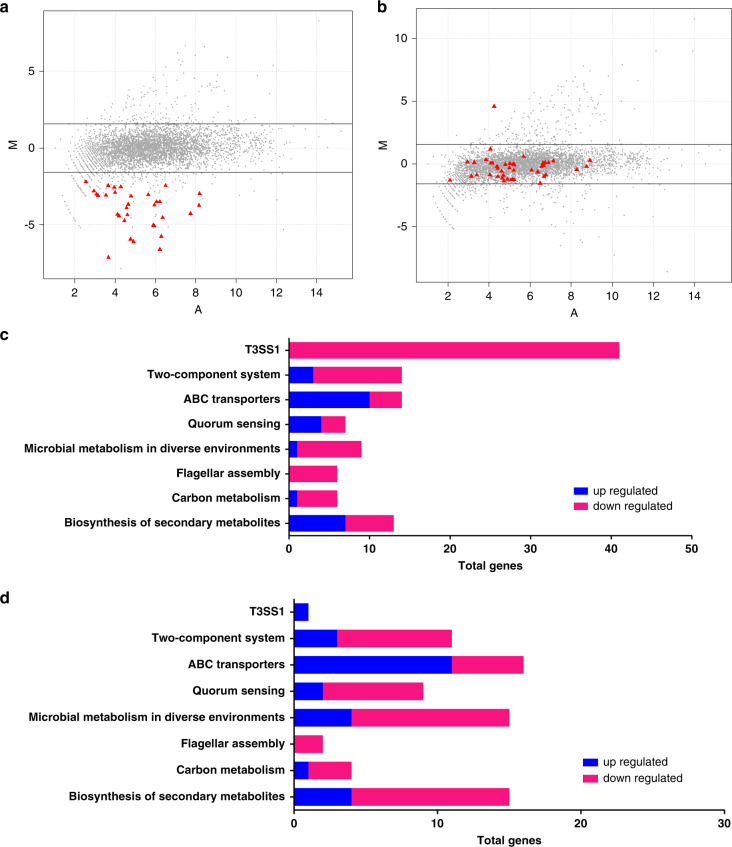
Nitrite and nitrate treatment alters global gene expression in *V.parahaemolyticus.* **a** MA plot of relative transcript abundance between nitrite-treated WT and untreated WT. Red triangles represent genes encoding T3SS1. **b** MA plot of relative transcript abundance between nitrate-treated WT and untreated WT. Red triangles represent genes encoding T3SS1. The Log_2_ of the ratio of abundance of each transcript between the two conditions (M in *y* axis) is plotted against the average log_2_ of abundance of that transcript in both conditions (A in *x* axis) (**a**, **b**). **c**, **d** Expression profiles of genes belonging to the indicated metabolic pathways. Red and blue colors show the genes that are reduced and elevated by threefold, respectively, after nitrite treatment (**c**) or nitrate treatment (**d**) compared to those under the untreated condition.

Because our previous studies have shown that T3SS1 overexpression leads to reduced colonization of *V. parahaemolyticu*s in the small intestine^30^, we reasoned that signals in the small intestine could dampen T3SS1 expression in order to sustain maximal colonization. We subsequently determined which signals can trigger the phosphorylation and activation of VbrK to repress *exsC* and T3SS1 expression. The intestinal environment is complex and composed of both mammalian and commensal-associated metabolites^36,37^. We performed a phosphorylation assay to determine whether some of these metabolites can trigger VbrK phosphorylation and activation. Our results showed that VbrK was phosphorylated in bacterial cells grown in the presence of sodium nitrite (Fig. 4a, lane 6). In contrast, other metabolic components, including butyrate, sodium nitrate, acetate, propionate, mannose, galactose, salicylic acid, succinate, mucin and hydrogen dioxide, did not trigger the phosphorylation of VbrK (Fig. 4a). Similarly, sodium nitrite, but not sodium nitrate, also triggered the phosphorylation of VbrR (Fig. 4b). Because nitrite can trigger the phosphorylation of VbrK and VbrR, we determined whether nitrite affects *exsC* expression. Quantitative RT-PCR results showed that *exsC* expression in the WT strain was significantly reduced after nitrite treatment, whereas the expression of *excC* in the Δ*vbrK* mutant was not inhibited by nitrite treatment (Fig. 4c). Further analysis showed that expression of T3SS1 structural (*vopD1* and *vp1662*) and effector (*vopS*) genes was also significantly reduced after nitrite treatment, while the expression of these genes in Δ*vbrK* mutant was not inhibited by nitrite treatment (Fig. 4d–f). These results demonstrated that nitrite could induce VbrK phosphorylation and repress *exsC* and T3SS1 gene expression. Since our previous studies have shown that β-lactam can also induce VbrK phosphorylation^34^, we determined if β-lactam can repress the expression of T3SS1 genes. The results showed that the expression of T3SS1 in presence of β-lactam was lower than that in the absence of β-lactam antibiotics, but was higher than that in the presence of nitrite. These results indicated that although β-lactam can inhibit the expression of T3SS1, the inhibitory effect is not as strong as nitrite (Supplementary Fig. 4). We further analyzed nitrite- and nitrate-mediated global gene expression by RNA-seq. The results showed that 150 genes were upregulated and 223 genes were downregulated by nitrite treatment (Fig. 5a). Similarly, 180 genes were upregulated and 192 genes were downregulated by nitrate treatment (Fig. 5b). KEGG pathway analysis showed that multiple cellular processes were affected by nitrite and nitrate treatment similarly (Supplementary Fig. 5a, b), which indicated that both nitrate and nitrite influence versatile cellular functions. Further analysis showed that the expression of the entire T3SS1 operon in the WT strain was significantly reduced after nitrite treatment (Fig. 5a, c). In contrast, the T3SS1 genes were not significantly affected by nitrate treatment (Fig. 5b, d), which is consistent to the results that nitrite, but not nitrate, could trigger the phosphorylation of VbrK and VbrR (Fig. 4a, b). Expression profile of genes belonging to several major metabolic pathways, such as TCS, ABC transporters, quorum sensing, and carbon metabolism were altered similarly by both nitrate and nitrite treatment (Fig. 5c, d). These results demonstrated both nitrite and nitrate had global effect on gene expression, but only nitrite affects T3SS1 gene expression.

### Nitrate reduces T3SS1 expression under anaerobic conditions

Our results showed that nitrite, but not nitrate, stimulates the phosphorylation of VbrK (Fig. 4a) and subsequently represses the expression of *exsC* and T3SS1 genes (Figs. 4c–f and 5a–d). It is possible that nitrate might not be reduced to nitrite under the aerobic conditions used in the above-described studies and thus cannot repress the expression of *exsC* and T3SS1 genes. We hypothesized that under anaerobic conditions, nitrate could be reduced to nitrite and would thus be able to repress the expression of *exsC*. The results showed that under aerobic conditions, the addition of nitrate did not significantly reduce the activity of the *excC* promoter in the WT strain (Supplementary Fig. 6a). In contrast, under anaerobic conditions, the addition of nitrate significantly reduced the activity of the *excC* promoter in the WT strain (Supplementary Fig. 6b). As a control, nitrate did not reduce the activity of the *exsC* promoter under both aerobic and anaerobic conditions in the Δ*vbrK* strain (Supplementary Fig. 6a, b). To further investigate whether the ability of nitrate to inhibit *excC* promoter activity under anaerobic conditions (Supplementary Fig. 6b) is due to its reduction to nitrite, we constructed a mutant strain (Δ*vpa1199*-*1201*) by deleting three predicted nitrate reductases (Vpa1199, Vpa1200 and Vpa1201) and determined whether the addition of nitrate could still reduce *exsC* expression in this mutant. The results showed that nitrate could not reduce *exsC* expression under both aerobic and anaerobic conditions in Δ*vpa1199*-*1201* (Supplementary Fig. 6a, b). As a control, deletion of these nitrate reductase had no effect on the ability of nitrite to inhibit *exsC* expression (Fig. 4c). These results strongly indicated that *V. parahaemolyticus* nitrate reductase converts nitrate to nitrite under anaerobic conditions and subsequently represses the expression of *exsC* and T3SS1 genes.

### Host-derived nitrite reduces T3SS1 expression during infection

**Fig. 6:**
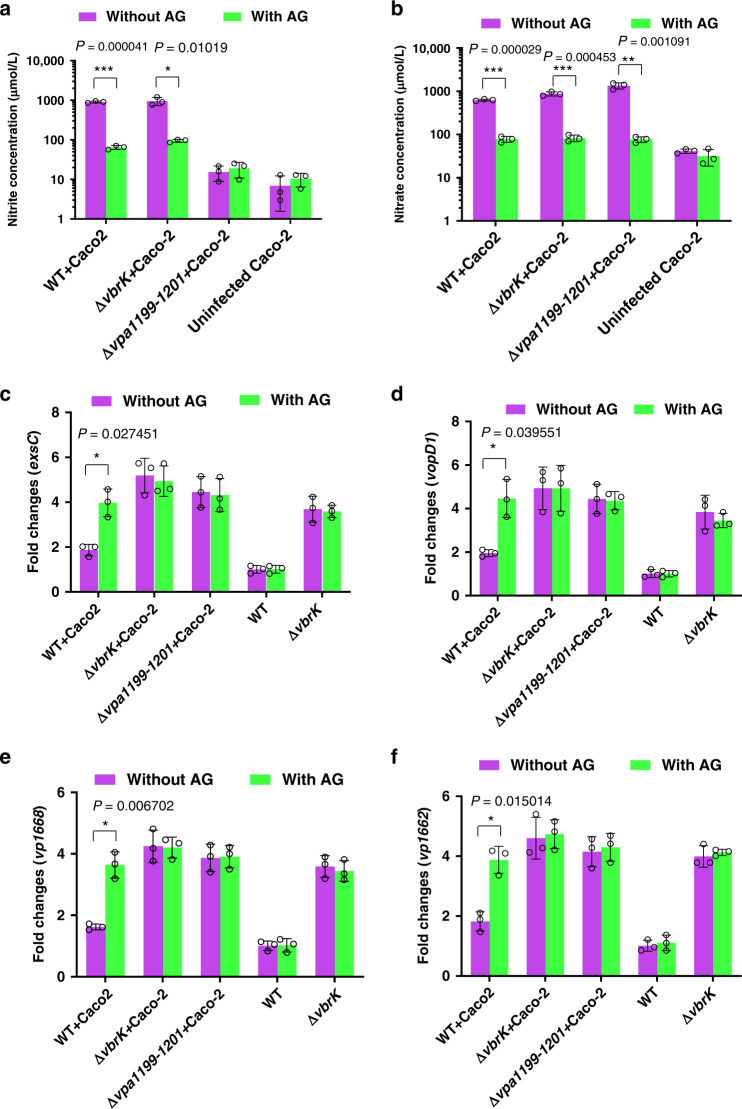
Host-derived nitrite represses T3SS1 gene expression during infection. **a** Nitrite concentration measured in the culture medium of Caco-2 cells infected with the indicated strains in the presence or absence of aminoguanidine (AG). All error bars represent mean ± standard deviation (*n* = 3 biologically independent experiments). **b** Nitrate concentration measured in the culture medium of Caco-2 cells infected with the indicated strains in the presence or absence of AG. All error bars represent mean ± standard deviation (*n* = 3 biologically independent experiments). **c**–**f** Quantitative RT-PCR analysis of T3SS1 gene expression in *V. parahaemolyticus* during infection. Caco-2 cells were infected with WT (WT + Caco-2), Δ*vbrK* (Δ*vbrK* + Caco-2) or *∆vpa1199-1201* (*∆vpa1199-1201* + Caco-2) and RNA was collected from the infected samples for qRT-PCR analysis of *exsC* (**c**), *vopD1* (**d**), *vp1668* (**e**) and *vp1662* (**f**). Transcripts of these genes in WT and Δ*vbrK* in the LB medium were also included as controls. Bars indicate average fold changes relative to WT under LB growth condition without AG. All error bars represent mean ± standard deviation (*n* = 3 biologically independent experiments). (*a*-*f*) Statistical significance was calculated using two-tailed multiple *t* test with Bonferroni correction. Asterisks indicate *P* values *P < 0.05, **P < 0.005, and ***P < 0.0005 (exact *P* value for each comparison was shown in the figures).

Bacterial infection can result in the upregulation of host inducible nitric oxide synthase (iNOS), which catalyzes the production of nitric oxide from arginine^38^. Nitric oxide can be quickly converted to nitrate through the intermediate product peroxynitrite^39^ and nitrate can be further reduced to nitrite during infection^36^. To determine the signal that represses T3SS1 gene expression during infection, we infected Caco-2 cells under anaerobic conditions with or without aminoguanidine (AG), a chemical that inhibits iNOS activity and thus reduces nitric oxide, nitrate and nitrite concentration. In the absence of AG treatment, nitrate and nitrite concentration in the infected cells (infection with WT or Δ*vbrK*) was much higher than that in the uninfected cells (Fig. 6a, b), which indicated that infection induces iNOS activity and increases the nitrate and nitrite levels. As expected, the nitrate and nitrite concentration in AG-treated Caco-2 cells after infection was significantly lower than that in the untreated cells (Fig. 6a, b), which indicated that AG inhibits iNOS activity and thus reduces the concentration of nitrate and nitrite. Furthermore, AG treatment significantly increased T3SS1 gene expression in the WT strain during infection under anaerobic conditions (Fig. 6c–f). In contrast, AG treatment did not significantly affect T3SS1 gene expression in Δ*vbrK* during infection under anaerobic conditions (Fig. 6c–f). As a control, AG itself did not affect T3SS1 gene expression in the WT or Δ*vbrK* strain in the absence of host cells (Fig. 6c–f). To ensure that inhibition of iNOS activity by AG is not due to its potential off target effects^40^, we further utilized N-(3-aminomethyl) benzyl acetamidine (1400 W), a potent and selective iNOS inhibitor, and the results showed that 1400 W treatment reduced nitrate and nitrite concentration, and increased T3SS1 gene expression (Supplementary Fig. 7a–d), consistent with what was observed when AG was used. These results demonstrated that host-derived nitric oxide/nitrate or nitrite can inhibit the expression of T3SS1 genes during infection in vitro. To further determine if nitric oxide/nitrate or nitrite represses T3SS1 gene expression, we infected Caco-2 cells with a nitrate reductase mutant, Δ*vpa1199*-*1201*. The results showed that the level of nitrate produced during infection with Δ*vpa1199*-*1201* was comparable to that produced during WT infection (Fig. 6b and Supplementary Fig. 7b), however, the expression of T3SS1 gene was repressed in WT (Fig. 6c–f, Supplementary Fig. 7c, d), but not in the Δ*vpa1199*-*1201* (Fig. 6c–f, Supplementary Fig. 7c, d). Because VbrK is still functional in Δ*vpa1199*-*1201* (Fig. 4c), these results indicated that nitrate or nitric oxide produced by iNOS during infection does not *S*-nitrosylate VbrK and repress T3SS1 gene expression. The results that infection with WT produced much higher nitrite than infection with Δ*vpa1199*-*1201* (Fig. 6a and Supplementary Fig. 7a) and T3SS1 gene expression is repressed in WT, but not in Δ*vpa1199*-*1201* (Fig. 6c–f, Supplementary Fig. 7c, d), strongly suggested that nitrite produced by bacterial nitrate reductase is responsible for VbrK *S*-nitrosylation and subsequent T3SS1 gene repression. The results that production of nitrite requires both host iNOS (Fig. 6a and Supplementary Fig. 7a) and bacterial nitrate reductase (Fig. 6a and Supplementary Fig. 7a) suggest that nitrite is produced in two steps: (1) host iNOS-catalyzed production of nitric oxide, which is quickly converted to nitrate; and (2) bacterial nitrate reductase-catalyzed conversion of nitrate to nitrite. To determine if T3SS2 and TDH have confounding effect, we used POR3 strain (both TDH and T3SS2 were mutated) as the parental strain in the culture infection assay, and the results showed that T3SS1 gene expression can be repressed in POR3, but not in POR3Δ*vbrK* (Supplementary Fig. 7e, f), consistent with the results when WT was used as parental strain (Fig. 6c–f), indicating that T3SS2 and TDH do not affect the ability of VbrK to sense nitrite and repress T3SS1 gene expression.

### Nitrite mediates the S-nitrosylation of VbrK in vitro

**Fig. 7:**
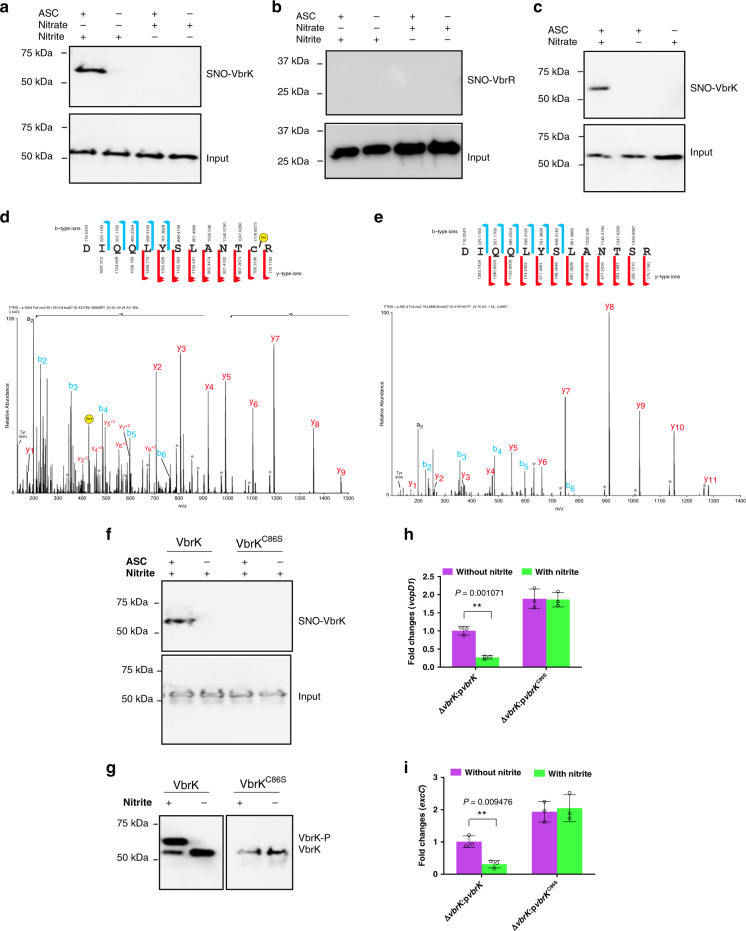
Nitrite triggers *S*-nitrosylation of VbrK. **a** Analysis of S-nitrosylated VbrK (SNO-VbrK) in *V. parahaemolyticus* (Δ*vbrK*:p*vbrK*_6xHis) cultured aerobically in the presence of nitrate or nitrite. **b** Analysis of S-nitrosylated VbrR (SNO-VbrR) in *V. parahaemolyticus* (Δ*vbrR*:p*vbrR*_6xHis) cultured aerobically in the presence of nitrate or nitrite. **c** Analysis of S-nitrosylated VbrK (SNO-VbrK) in *V. parahaemolyticus* (Δ*vbrK*:p*vbrK*_6xHis) cultured anaerobically in the presence or absence of nitrate. Assays in the absence of sodium ascorbate (ASC) was included as a negative control (**a**–**c**). Samples were blotted with anti-His antibody (**a**–**c**). **d** Mass spectrometry (MS/MS) of a biotin-charged VbrK peptide derived from *V. parahaemolyticus* (Δ*vbrK*:p*vbrK*_6xHis) cultured in the presence of nitrite. **e** Mass spectrometry (MS/MS) of VbrK^C86S^ peptide derived from *V. parahaemolyticus* (Δ*vbrK*:p*vbrK*^C86S^_6xHis) cultured in the presence of nitrite. **f** Analysis of VbrK and VbrK^C86S^ *S*-nitrosylation in *V. parahaemolyticus* (Δ*vbrK*:p*vbrK*_6xHis and Δ*vbrK*:p*vbrK*^C86S^_6xHis) cultured in the presence of nitrite. **g** Phosphorylation of VbrK and VbrK^C86S^ in *V. parahaemolyticus* (Δ*vbrK*:p*vbrK*_6xHis and Δ*vbrK*:p*vbrK*^C86S^_6xHis) cultured in the presence or absence of nitrite. Quantitative RT-PCR analysis of *vopD1* (**h**) and *exsC* (**i**) in the presence or absence of nitrite. Bars indicate average fold changes of *vopD1* and *exsC* compared to that in Δ*vbrK*:p*vbrK* without nitrite treatment. All error bars represent mean ± standard deviation (*n* = 3 biologically independent experiments). Statistical significance was calculated using two-tailed multiple *t* test with Bonferroni correction. Asterisks indicate *P* values *P < 0.05, **P < 0.005, and ***P < 0.0005.

We subsequently explored how nitrite induces the phosphorylation of VbrK (Fig. 4a) and thereby reduces T3SS1 gene expression (Figs. 4c–f, 5a, c). *S*-nitrosylation of cysteine residue of proteins following exposure to nitric oxide has been observed in both eukaryotic and prokaryotic organisms, and this modification influences the functions of many proteins^41,42^. We hypothesized that nitrite can induce the *S*-nitrosylation of VbrK, which results in its subsequent phosphorylation. We performed a biotin-switch assay to determine the *S*-nitrosylation of recombinant VbrK. Under aerobic growth condition, the *S*-nitrosylation of VbrK was not detected after nitrate treatment (Fig. 7a), whereas VbrK was *S*-nitrosylated after nitrite treatment (Fig. 7a). In contrast, VbrR was not *S*-nitrosylated in the presence of either nitrite or nitrate (Fig. 7b). Under anaerobic growth condition, *S*-nitrosylation of VbrK was observed after nitrate treatment (Fig. 7c), consistent with the results that nitrate could inhibit T3SS1 gene expression under anaerobic condition (Supplementary Fig. 6b), but not under aerobic condition (Supplementary Fig. 6a). VbrK contains seven cysteine residues, and we determined which cysteine residue is involved in the *S*-nitrosylation of VbrK. Recombinant VbrK from *V. parahaemolyticus* grown aerobically in the presence of nitrite was processed using the biotin-switch assay and analyzed by mass spectrometry. At least three independent experiments showed that C86 was consistently *S*-nitrosylated when *V. parahaemolyticus* was grown in the presence of nitrite (Fig. 7d). The *S*-nitrosylation of this residue substituted with serine (Fig. 7e) was not detected in the presence of nitrite (Fig. 7e, f). These results demonstrated that nitrite could trigger the *S*-nitrosylation of VbrK at C86.

### The S-nitrosylation of C86 is essential for VbrK phosphorylation and T3SS1 gene repression

Because VbrK could be *S*-nitrosylated (Fig. 7a, d) and phosphorylated (Fig. 4a) after treatment with nitrite, we explored whether the *S*-nitrosylation of VbrK is essential for VbrK phosphorylation. In the absence of nitrite, neither VbrK nor VbrK^C86S^ was phosphorylated (Fig. 7g). After nitrite treatment, WT VbrK was phosphorylated, whereas VbrK^C86S^ was not phosphorylated (Fig. 7g), which indicated that *S*-nitrosylation is important for VbrK phosphorylation after nitrite treatment. To assess the significance of VbrK *S*-nitrosylation in repressing T3SS1 gene expression, we evaluated the expression of T3SS1 genes in Δ*vbrK*:p*vbrK* and Δ*vbrK*:p*vbrK*^C86S^. T3SS1 genes (*excC* and *vopD1*) expression in Δ*vbrK*:p*vbrK* was significantly reduced after nitrite treatment (Fig. 7h, i), whereas the expression of these two genes in Δ*vbrK*:p*vbrK*^C86S^ was not significantly changed after nitrite treatment (Fig. 7h, i). These results indicated that the *S*-nitrosylation of VbrK is essential for the nitrite-mediated repression of T3SS1 gene expression.

### Nitrite-mediated S-nitrosylation of VbrK is essential for downregulation of the inflammatory response both in vitro and in vivo

**Fig. 8:**
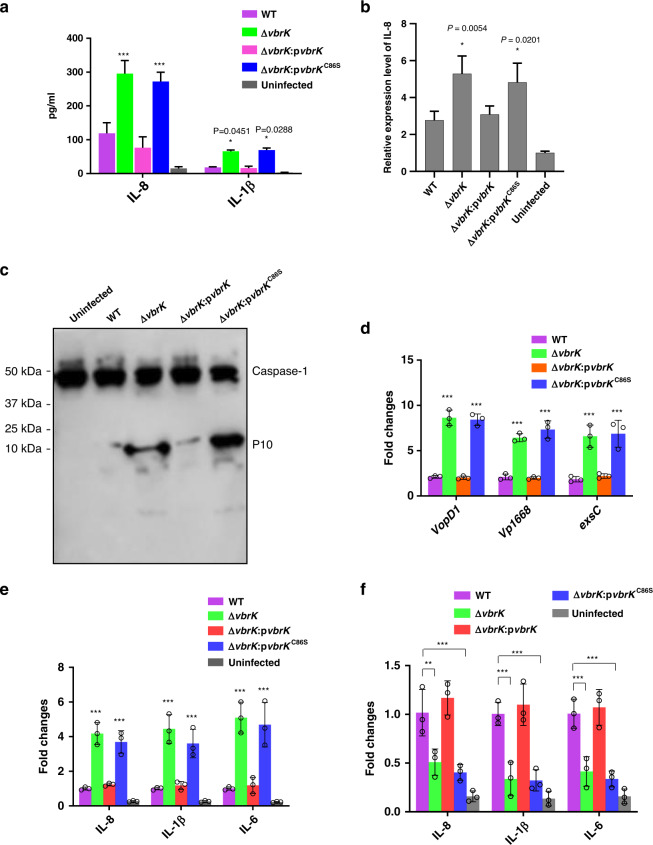
*S*-nitrosylation of VbrK is important for dampening the proinflammatory cytokines both in vitro and in vivo. **a** IL-8 and IL-1β concentration in the culture medium of uninfected Caco-2 cells or Caco-2 cells infected with the indicated strains. All error bars represent mean ± standard deviation (*n* = 3 biologically independent experiments). Statistical significance was calculated using two-way ANOVA with Bonferroni correction. Asterisks indicate *P* values *P < 0.05, **P < 0.005, and ***P < 0.0005 compared to the cells infected with WT. **b** Relative expression of IL-8 in Caco-2 cells infected with indicated strains. Bars indicate average fold changes of IL-8 transcript relative to that in the uninfected cells. All error bars represent mean ± standard deviation (*n* = 3 biologically independent experiments). Statistical significance was calculated using one-way ANOVA with Bonferroni correction. Asterisks indicate *P* values *P < 0.05, **P < 0.005, and ***P < 0.0005 compared to cells infected WT. **c** Caspase-1 cleavage in Caco-2 cells infected with the indicated strains. **d** Quantitative RT-PCR analysis of T3SS1 genes in the infant rabbits infected with the indicated strains for 18 h. Bars represent fold changes of T3SS1 genes compared to the expression of respective genes under in vitro LB growth condition. All error bars represent mean ± standard deviation (*n* = 3 biologically independent experiments). Statistical significance was calculated using two-way ANOVA with Bonferroni correction. Asterisks indicate *P* values *P < 0.05, **P < 0.005, and ***P < 0.0005 compared to those in WT during infection. **e** Quantitative RT-PCR analysis of cytokine genes in the infant rabbits infected with the indicated strains for 18 h. Bars represent fold changes of the cytokines compared to the expression of the respective cytokines in the rabbits infected with WT. All error bars represent mean ± standard deviation (*n* = 3 biologically independent experiments). Statistical significance was calculated using two-way ANOVA with Bonferroni correction. Asterisks indicate *P* values *P < 0.05, **P < 0.005, and ***P < 0.0005 compared to the cytokine expression in WT-infected rabbits. **f** Quantitative RT-PCR analysis of cytokine genes in the infant rabbits infected with the indicated strains for 38 h. Bars represent fold changes compared to the expression of these cytokines in rabbits infected with WT. All error bars represent mean ± standard deviation (*n* = 3 biologically independent experiments). Statistical significance was calculated using two-way ANOVA with Bonferroni correction. Asterisks indicate *P* values *P < 0.05, **P < 0.005, and ***P < 0.0005 compared to the cytokine expression in WT-infected rabbits.

Previous studies have shown that the *V. parahaemolyticus* T3SS1 can trigger inflammasome activation in macrophages in vitro^27^. Furthermore, VopQ (Vp1680), a T3SS1 effector, plays an important role in inducing IL-8 secretion during infection with intestinal epithelial cells in vitro^28^. These results suggest that T3SS1 plays an important role in triggering the inflammatory response. Based on our results that the *S*-nitrosylation of VbrK inhibits T3SS1 gene expression (Fig. 7h, i), we hypothesized that the *S*-nitrosylation of VbrK also dampens the inflammatory response during infection. To determine whether *S*-nitrosylation-mediated T3SS1 gene repression leads to the downregulation of inflammation in vitro, we infected intestinal epithelial cells, Caco-2 cells, under anaerobic conditions for 4 h and then measured the secretion of proinflammatory cytokines IL-8 and IL-1β. It is worth to note that cytoxicity of *V. parahaemolyticus* was minimal under the anaerobic condition (WT and Δ*vbrK* mutant killed ~25% and 30% of the Caco-2 cells, respectively, for the time period assayed in this study) (Supplementary Fig. 8), thus cytotoxicity would not dramatically confound the interpretation of cytokine secretion results. The results showed that the concentrations of IL-8 and IL-1β in the supernatant of Caco-2 cells infected with Δ*vbrK* were significantly higher than those observed after infection with the WT strain (Fig. 8a). The complementation of Δ*vbrK* with VbrK (Δ*vbrK*:p*vbrK*), but not VbrK^C86S^ (Δ*vbrK*:p*vbrK*^C86S^), restored the concentrations of proinflammatory cytokines to the concentrations found in cells infected with WT (Fig. 8a). Similarly, transcripts of IL-8 in cells infected with Δ*vbrK* and Δ*vbrK*:p*vbrK*^C86S^ were significantly higher than those infected with WT and Δ*vbrK*:p*vbrK* (Fig. 8b). Caspase-1 was highly activated in the cells infected with Δ*vbrK*:p*vbrK*^C86S^ or Δ*vbrK*, but not in WT or Δ*vbrK*:p*vbrK*-infected cells (Fig. 8c). These results demonstrated that the nitrite-mediated *S*-nitrosylation of VbrK is important for dampening the inflammatory response during infection in vitro. We then determined whether the role of the *S*-nitrosylation of VbrK in the modulation of the inflammatory response could be recapitulated in vivo in an infant rabbit infection model. As expected, the expression levels of T3SS1 genes in the Δ*vbrK-* and Δ*vbrK*:p*vbrK*^C86S^-infected rabbits were significantly higher than those in the WT- and Δ*vbrK*:p*vbrK*-infected rabbits (Fig. 8d) following 18 h of infection. The results further showed that infant rabbits infected with Δ*vbrK* for 18 h had significantly higher IL-6, IL-1β, and IL-8 expression levels than those infected with the WT strain (Fig. 8e). The complementation of Δ*vbrK* with VbrK (Δ*vbrK*:p*vbrK*), but not VbrK^C86S^ (Δ*vbrK*:p*vbrK*^C86S^), restored the expression levels of IL-6, IL-1β, and IL-8 to the expression levels in rabbits infected with the WT strain (Fig. 8e). Interestingly, after 38 h infection, rabbits infected with Δ*vbrK* or Δ*vbrK*:p*vbrK*^C86S^ had significantly lower expression of cytokines than those infected with WT or Δ*vbrK* (Fig. 8f). These results demonstrated that the *S*-nitrosylation of VbrK is also important for dampening the expression of proinflammatory cytokines in vivo at the early stage of infection, but not at later stage of infection.

### The S-nitrosylation of VbrK contributes to bacterial colonization in the small intestine and virulence

**Fig. 9:**
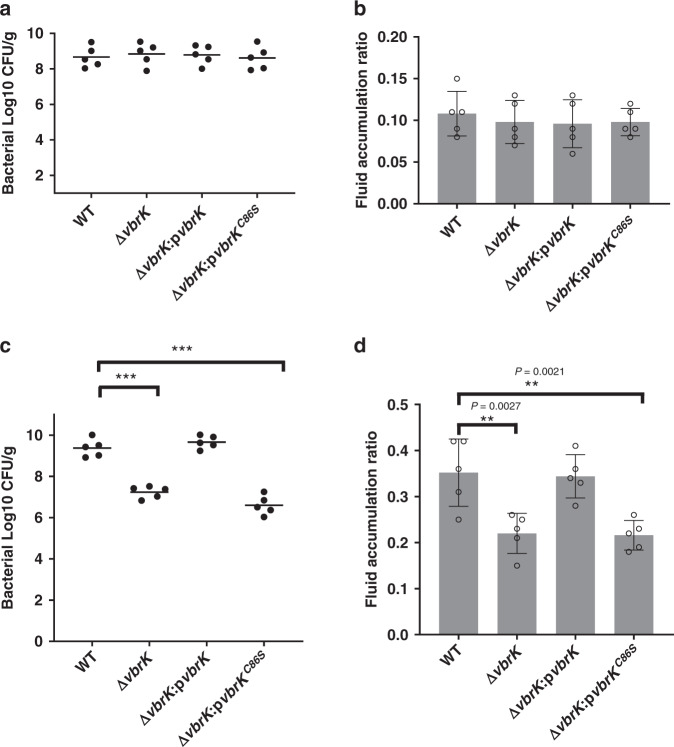
*S*-nitrosylation is important for colonization and virulence after 38 h of infection. **a** Bacterial CFU in the infant rabbits (*n* = 5 biologically independent rabbits) infected with the indicated strains for 18 h. **b** Fluid accumulation in the infant rabbits (*n* = 5 biologically independent rabbits) infected with the indicated strains for 18 h. All error bars represent mean ± standard deviation. **c** Bacterial CFU in the infant rabbits (*n* = 5 biologically independent rabbits) infected with the indicated strains for 38 h. All error bars represent mean ± standard deviation. Statistical significance was calculated using two-way ANOVA with Bonferroni correction. Asterisks indicate *P* values *P < 0.05, **P < 0.005, and ***P < 0.0005. **d** Fluid accumulation in the infant rabbits (*n* = 5 biologically independent rabbits) infected with the indicated strains for 38 h. All error bars represent mean ± standard deviation. Statistical significance was calculated using two-way ANOVA with Bonferroni correction. Asterisks indicate *P* values *P < 0.05, **P < 0.005, and ***P < 0.0005.

Because the *S*-nitrosylation of VbrK led to the downregulation of proinflammatory cytokines at the early stage of infection in vivo, we determined whether this downregulation of inflammation contributes to bacterial colonization and fluid accumulation. No significant difference in intestinal colonization after 18 h of infection was found between the WT and mutants (Fig. 9a), and the fluid accumulation ratio also showed no significant difference between the WT and mutants (Fig. 9b). However, after 38 h of infection, the bacterial CFUs in the Δ*vbrK-* and Δ*vbrK*:p*vbrK*^C86S^-infected rabbits were significantly lower than those in the WT- and Δ*vbrK*:p*vbrK*-infected rabbits (Fig. 9c). Similarly, the fluid accumulation levels in the Δ*vbrK-* and Δ*vbrK*:p*vbrK*^C86S^-infected rabbits were significantly lower than those in the WT- and Δ*vbrK*:p*vbrK*-infected rabbits after 38 h of infection (Fig. 9d). These results indicated that the dampening of the inflammatory response mediated by the *S*-nitrosylation of VbrK at the early stage of infection is associated with robust colonization in the small intestine and the high virulence of *V. parahaemolyticus* at the later stage of infection.

## Discussion

**Fig. 10:**
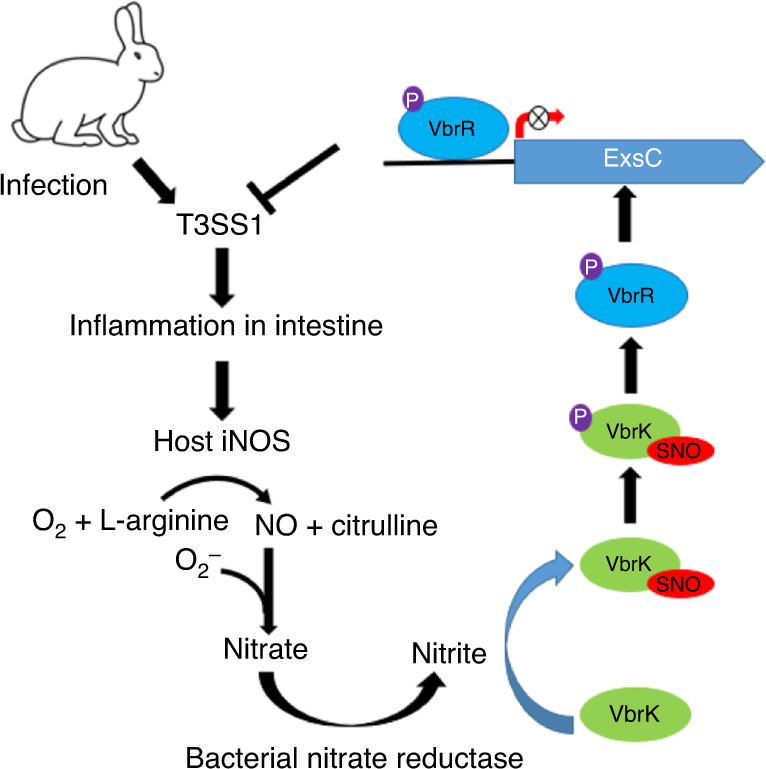
A model describing S-nitrosylation of VbrK mediates virulence of *V*. *parahaemolyticus*. During the early stage of infection, T3SS1 (which is upregulated in vivo compared to in vitro) stimulates the production of proinflammatory cytokines in the intestinal epithelial cells. These proinflammatory cytokines or bacterial infection itself trigger the expression of iNOS in intestinal epithelial cells, which catalyzes the production of NO from l-arginine. NO is rapidly converted to nitrate through the intermediate product peroxynitrite. Nitrate is reduced to nitrite by nitrate reductase in *V. parahaemolyticus*. Nitrite subsequently induces *S*-nitrosylation of VbrK, leading to its phosphorylation and phosphate transfer to VbrR. VbrR can bind *exsC* promoter and such binding results in the decreased expression of *exsC*, which ultimately leads to the decreased expression of T3SS1. Reduction in T3SS1 expression could reduce the production of proinflammatory cytokine, leading to robust colonization and virulence. Mutation of the *S*-nitrosylation site C86 of VbrK leads to the inability of nitrite to reduce T3SS1 gene expression and thus stronger proinflammatory response was induced. Strong proinflammatory response could reduce bacterial colonization and virulence at the later stage of infection.

In this study, we identified an unprecedented mechanism of HK activation and such activation is important for dampening the host inflammatory response and promoting the virulence of *V. parahaemolyticus*. This conclusion was demonstrated by (1) VbrK, a HK previously shown to be activated by β-lactam antibiotics, can be *S*-nitrosylated by host-derived nitrite (Fig. 7a, d); (2) mutation of the *S*-nitrosylation site results in the deficiency of nitrite-mediated VbrK activation (Fig. 7g); and (3) mutation of *S*-nitrosylation site leads to higher T3SS1 gene expression (Fig. 7h, i), higher inflammatory response at early stage of infection and reduced colonization and virulence at later stage of infection (Figs. 8 and 9). Based on these results, we propose a model (Fig. 10) in which *S*-nitrosylation of VbrK serves as a feedback mechanism that decreases *V. parahaemolyticus* T3SS1 gene expression and thereby reduces host inflammatory response in order to escape the host immune defense and achieve robust virulence.

During infection, enteric pathogens initially stimulate inflammation in the gastrointestinal tract^43^, and this proinflammatory response is beneficial for the growth of enteric pathogens and their colonization of the GI tract^44^. However, uncontrolled inflammation can result in the elimination of pathogens^45,46^. Therefore, many enteric pathogens are equipped with multiple strategies to control the host innate inflammatory response^47^. Many of these strategies are associated with secreted bacterial effectors that are translocated into host cells to interfere with the host signaling pathways responsible for the induction of proinflammatory cytokines^47^. In this study, we identified a mechanism in which the downregulation of T3SS1 (a proinflammatory response stimulator) contributes to the repression of the innate immune response and thus robust colonization of *V. parahaemolyticus* in the GI tract. These findings were demonstrated by the results that mutation in a negative regulator of T3SS1, VbrK, resulted in a marked decrease in the colonization and fluid accumulation in the small intestine of infant rabbits (Fig. 9). Our results that Δ*vbrK* triggers higher levels of inflammation at the early stage of infection (Fig. 8), and lower levels of colonization at the late stage of infection (Fig. 9) support a mechanism in which the downregulation of virulence factor expression promotes virulence by dampening the inflammatory response. The utilization of T3SS1 downregulation to control inflammation to the appropriate level to sustain robust colonization highlights the importance of the fine-tuned regulation of virulence factors in the inflammatory response and immune evasion. Previous transcriptomic studies have shown that *V. parahaemolyticus* during infection in infant rabbits exhibit upregulated T3SS1 gene expression compared to that in culture medium^48^, which is consistent with our quantitative RT-PCR (Fig. 8d) and suggests that unidentified host factors trigger T3SS1 gene expression in vivo. The finding that T3SS1 can trigger the production of proinflammatory cytokines in vitro^28^ is contradictory to the results from a previous study, which showed that the deletion of T3SS1 does not affect proinflammatory cytokine production at later stages of infection in vivo^11^. We suspect that this could be due to the reason that, at a later stage of infection, other strong inflammation stimulators, e.g., LPS and flagellin, dominates the proinflammatory cytokines production even though T3SS1 is not present. For example, flagellin activates TLR5 on the basolateral surface only when the tight junctions were open and bacteria gain access to TLR5 to activate IL-8^49,50^, indicating that flagellin promote inflammatory response when the integrity of intestinal epithelium is compromised possibly at the later stage of infection. Overall, we conclude that at the early stage of infection, T3SS1 gene expression is upregulated to promote inflammation, which results in the production of nitrate and nitrite (Fig. 10). Subsequently, nitrite dampens the expression of T3SS1 to avoid excessive inflammation, which would promote immune evasion and enhance colonization and virulence at the late stage of infection (Fig. 9). The results that at later stage of infection inflammatory cytokines were lower in Δ*vbrK* or Δ*vbrK*:p*vbrK*^C86S^-infected animals compared to WT-infected animals (Fig. 8e) may be due to the fact that Δ*vbrK* or Δ*vbrK*:p*vbrK*^C86S^ colonized at lower level at later stage of infection (Fig. 9c) and thus the inflammation was reduced.

Inflammatory cytokines upregulate the expression of iNOS^51^, which catalyzes the conversion of l-arginine to nitric oxide in intestinal epithelial cells^51,52,39^. Nitric oxide reacts with superoxide formed during inflammation to produce peroxinitrite, which quickly decomposes to nitrate, a reaction catalyzed by carbon dioxide^39,52^. Enteric pathogens, but not commensals, harbor nitrate reductase and can thus convert nitrate to nitrite, which provides a growth advantage to pathogens over commensals in an anaerobic environment^53^. Here, we found that nitrite produced during infection serves as a signal to dampen the expression of T3SS1 (Fig. 6 and Supplementary Fig. 7) and thus provides negative feedback to reduce the inflammatory response. By reducing the inflammatory response, *V. parahaemolyticus* could potentially achieve robust colonization and virulence in vivo at later stages. Our results demonstrate that nitrate does not reduce the expression of T3SS1 under aerobic condition (Supplementary Fig. 6a) but is able to inhibit the expression of T3SS1 under anaerobic condition (Supplementary Fig. 6b), which indicated that anaerobic conditions promote the reduction of nitrate to nitrite. This finding is consistent with those obtained in previous studies, which showed that a high concentration of nitrite was only obtained with culture medium containing low oxygen concentrations (<2%)^54^. The results that mutants lacking nitrate reductases lost their ability to inhibit T3SS1 gene expression even under anaerobic conditions (Supplementary Fig. 6b) further demonstrated that nitrate needs to be reduced to nitrite before it can exert its role in repressing T3SS1 gene expression. Although nitric oxide has been shown to influence virulence gene expression^55^, the effect of nitrite, which is the donor of nitric oxide, on virulence gene expression has not been reported. Thus, in addition to the role of nitrite in providing a growth advantage to enteric pathogens over commensals, our results demonstrated that nitrite also serves as a signal that modulates T3SS1 gene expression in *V. parahaemolyticus* to avoid an excessive immune response and achieve robust colonization in vivo.

TCSs regulate gene expression in response to diverse environments^32,56^. Environmental cues activate HKs of TCS either directly or indirectly. For direct activation, small molecules can directly bind to the sensor domain of HK, which results in phosphorylation of the catalytic domain of HK and subsequent transfer of a phosphate to the RR^57^. The only known HK that can be activated by nitrate is NarQ, which can be activated through direct binding with nitrate^58^. Here, we provide the first demonstration that HK can be activated by nitrite-mediated *S*-nitrosylation. *S*-nitrosylation can occur by the transfer of a NO moiety to cysteine thiols in the target protein. As a reservoir of NO, nitrate can efficiently cause the *S*-nitrosylation of transcription regulator OxyR in *Escherichia coli* in a nitrate reductase-dependent manner^59^, which indicates that nitrate needs to be reduced to nitrite to cause *S*-nitrosylation. We showed that the addition of nitrite, but not nitrate, induces the *S*-nitrosylation of VbrK under aerobic conditions (Fig. 7a). However, during bacterial growth under anaerobic conditions, nitrate can also induce *S*-nitrosylation (Fig. 7c). Based on a previous study showing that nitrite production can be significantly increased during growth under low-oxygen conditions compared to high-oxygen conditions^54^, we hypothesize that under anaerobic conditions, nitrate is converted into nitrite, leading to the *S*-nitrosylation of VbrK and inhibition of T3SS1 gene expression. This hypothesis is supported by the results that T3SS1 gene expression is not inhibited by nitrate in the nitrate reductase mutant (Supplementary Fig. 6b). There are several mechanisms that nitrite can cause protein *S*-nitrosylation. Nitrous acid derived from nitrite can be protonated and lose water to generate the nitrosonium ion, which directly *S*-nitrosylates target proteins. Nitrite could be reduced to nitric oxide^60^, which can result in *S*-nitrosylation through Hcp enzymatic reaction^61^ or through oxidation of nitric oxide into nitrosonium ion by transition metals^62^. Nitrite added to the growth medium can also produce dinitrogen trioxide (N_2_O_3_), a potent nitrosant that nitrosylates the thiol group^63,64^. Direct nitrosylation by nitrite has also been observed in previous studies, but the underlying mechanisms remain unknown^65,66^. It is less likely that VbrK is *S*-nitrosylated by N_2_O_3_ because N_2_O_3_ is typically produced by nitrite under acidic conditions^67^. Yet, we showed that nitrite can strongly trigger *S*-nitrosylation under neutral conditions used in this study (Fig. 7a, d). Furthermore, T3SS1 gene expression in a mutant that lacks nitrate reductases is not repressed during infection (Fig. 6c–f) even though nitrate is produced at high level (Fig. 6b), indicating that nitric oxide and nitrate produced in the host cells by iNOS-catalyzed reaction does not *S*-nitrosylate VbrK. These results further indicate that *S*-nitrosylation of VbrK and subsequent T3SS1 repression during infection are dependent on the nitrate reductase-mediated conversion of nitrate (produced by iNOS-catalyzed reaction during infection) to nitrite. It remains to be determined whether or not nitrite needs to be further converted to nitric oxide or nitrosonium ion to *S*-nitrosylate VbrK. Although the RR of TCS has been shown to be a target of *S*-nitrosylation^67^, HK activation by *S*-nitrosylation has not been reported. Thus, our results that HK can be activated via *S*-nitrosylation represent an unprecedented mechanism through, which environmental cues are sensed and processed by bacterial pathogens.

Mutation of the *S*-nitrosylation site (C86S) of VbrK abolished the ability of *V. parahaemolyticus* to maximally colonize the small intestine (Fig. 9), which indicated that *S*-nitrosylation plays an important role in pathogenesis. The results that *S*-nitrosylation is also essential for dampening T3SS1 gene expression and the inflammatory response suggest that *S*-nitrosylation influences virulence potentially through subduing inflammation. *S*-nitrosylation also influences bacterial virulence through other mechanisms. For example, the transcription factor AgrA in *Staphylococcus* can be *S*-nitrosylated, which results in reductions in its abilities to bind to target gene promoters and induce the production of QS-mediated toxin and thereby a decreased virulence^55^. The *S*-nitrosylation of bacterial effector proteins can also inhibit phosphothreonine lyase activity, and therefore, its ability to interfere with host MAPK signaling is inhibited^68^. *Clostridium difficile* toxins are *S*-nitrosylated by the infected host, and *S-*nitrosylation attenuates virulence by inhibiting toxin self-cleavage and cell entry^69^. However, the signals that triggered the *S*-nitrosylation of the target proteins investigated in the above-mentioned studies have not been clearly defined. We demonstrated that the nitrite-mediated *S*-nitrosylation of VbrK is required for the subsequent phosphorylation of VbrK, which highlights the importance of crosstalk between different types of protein modifications in regulating bacterial virulence. The fact that β-lactam antibiotics can also activate VbrK and reduce T3SS1 gene expression (Supplementary Fig. 4) suggest that utilization of this class of antibiotics could potentially enhance *V. parahaemolyticus* virulence in the event that nitrite synthesis pathway is disrupted in the host.

In conclusion, host-derived induces *S*-nitrosylation of a HK, VbrK, which is essential for its phosphorylation and the subsequent transfer of a phosphate group to its RR, VbrR, to inhibit T3SS1 gene expression. The *S*-nitrosylation of VbrK provides negative feedback to avoid excessive inflammation, which would otherwise decrease bacterial colonization and the ability to cause fluid accumulation in the small intestine (Fig. 10). The presence of C86 in VbrK homologs in all *Vibrio* species^34^ indicates that *S*-nitrosylation potentially represents a conserved mechanism of HK activation in *Vibrio* species and potentially other bacterial species.

## Methods

### Bacterial strains, plasmids, and growth conditions

The bacterial strains and plasmids used in this study are listed in Supplementary Table 1. *E. coli* SM10 *λ*pir strains were used for conjugation with *V. parahaemolyticus*. The 6xHis*-exsC* strain was constructed by integrating a 6xHis tag at the C-terminus of *exsC* using the suicide plasmid pDM4^22^. DNA sequences for 6xHis were inserted before the stop codon of the *exsC* and cloned into pDM4. Following two crossovers, clones with 6xHis sequences inserted into the genome were selected. The deletion mutant of *V. parahaemolyticus* and the complement strains were generated using pDM4 and pMMB207 vectors, respectively^21^. Full length sequences of the gene were amplified and cloned downstream of the Ptac promoter in pMMB207 for complementation. All strains were grown at 37 °C in Luria-Bertani (LB) media. Antibiotics were used at the following concentrations: carbenicillin at 50 μg/ml and chloramphenicol at 25 μg/ml. Caco-2 cells were maintained in DMEM supplemented with 10% fetal bovine serum at 37 °C in an atmosphere containing 5% CO_2_. To generate anaerobic condition, bacteria were plated on LB agar and placed in a sealed box containing AnaeroPackTM (Thermo Fisher Scientific, Waltham, MA). Caco-2 cells were infected in six-well plate and placed in the anaerobic box for 4 h before subsequent experiments were performed.

### Experimental animals

Animal experiments were approved by the Institutional Animal Care and Use Committee (IACUC) of University of Connecticut (Protocol #A13-060). Infant rabbits were infected at the dose of 10^9^ CFU orally^11^. Colonization was measured by homogenizing the intestine tissues and enumerating the CFU in 1 g of tissues. Fluid accumulation in the small intestine were measured by weighing the small intestine before and after the fluid was removed^11^.

### Construction of plasmids

Primers used in this study are listed in Supplementary Table 2. VbrR open reading frame with a 6xHis tag at the C-terminus was amplified and cloned to pMMB207 for expression and purification. Allelic exchange plasmids were constructed by ligating the upstream and downstream sequences of the gene and inserting into the suicide vector pDM4. LacZ plasmid was constructed by inserting *exsC* promoter sequences into pDM8 to create fusion between P_*exsC*_ and *lacZ* gene (P_*exsC*_-lacZ). Similarly, *exsC* promoter without VbrR-binding site (P_*exsC*Δ50_-lacZ) was inserted into pDM8.

### Construction of bacterial strains

Deletion mutations were constructed by allelic exchange using pDM4 that harbors the upstream and downstream sequences of genes^21^. Strains overexpressing predicted sigma factor were constructed by introducing pMMB207 plasmid harboring the corresponding sigma factor gene into the WT. Integration of 6xHis tag at the C-terminus of *exsC* in the chromosome was performed by double crossovers using sucrose^22^. Strains used to measure lacZ activity were constructed by introducing pDM8 harboring P_*exsC*_-lacZ or P_*exsC*Δ50_-lacZ into WT, ∆*vbrK*, ∆*vbrR*, ∆*vbrK*∆*vp2210* and Δ*vpa1199-1201*.

### Bacterial protein secretion assay

The supernatant of *V. parahaemolyticus* was prepared by centrifugation of the culture and precipitation using trichloroacetic acid^21^. The pellets were normalized to equal amounts based on the OD_600_ values. The proteins were separated by 12% SDS-PAGE and subsequently transferred to PVDF membranes, and the membranes were then incubated with the VopD1 antibody at 1:2000 dilution^21^. HRP-conjugated anti-mouse IgG (1:1000) was then added, and the blots were visualized using the ECL reagent. RNAP detected by mouse monoclonal Anti-RNA polymerase antibody (1:1000) (BioLegend, San Diego, CA) was used as a sample processing control to ensure that equal amount of protein was loaded. To analyze the ExsC expression, whole cell lysates of *V. parahaemolyticus* were separated by 12% SDS-PAGE, and western blotting was performed using HRP-conjugated anti-His antibody (1:1000) (R&D System, Minneapolis, MN). The anti-His antibody was used to measure the expression of ExsC because a 6xHis tag was integrated at the C-terminus of *exsC* in the genome.

### RT-PCR, quantitative RT-PCR, and RNA-seq

The overnight bacterial culture was diluted 1:100 with fresh LB medium. After 4 h of incubation with shaking in the presence or absence of nitrite, total RNA was isolated using an RNA Easy Kit (Qiagen, Valencia, CA, USA). Total RNA was treated with DNase I (Promega, Madison, WI) for 30 min to remove the genomic DNA. Subsequently, 0.5 μg of RNA was used for reverse transcription. SYBR Green qPCR Mix (Biomake, Houston, TX) was used for quantitative PCR with the primers listed in Supplementary Table 2. All T3SS1 gene transcripts were normalized to the DNA gyrase subunit B (*gyrB*) gene and relative fold change was determined using the ΔΔCt method^70^. ΔCt values (the difference between Ct value of T3SS1 gene and gyrB) were first measured and then ΔΔCt values (the difference of ΔCt value between different conditions) were calculated. RNA-seq was performed by Genewiz (South Plainfield, NJ) and data analysis was performed using Rockhopper program 2.0.3 and edgeR program 3.30.3. Transcriptome analysis of WT in the presence or absence of 500 μM nitrite or nitrate was performed to identify genes regulated by nitrite and nitrate, respectively. The differentially expressed genes were determined with fold change ≥ 3. The genome of *Vibrio parahaemolyticus* RIMD2210633 (https://www.genome.jp/kegg-bin/show_organism?org=vpa) was used as reference for annotation.

### Electrophoretic mobility shift assay (EMSA)

The EMSAs were performed using purified VbrR or Vp2210 proteins^71^. The purified 6xHis-tagged VbrR protein (purified using Δ*vbrR:pvbrR_6xHis* strain) was incubated with different FAM-labeled DNA probes in a volume of 20 μl. Excessive poly(dI:dC) (Sigma-Aldrich, St. Louis, MO) was added to inhibit nonspecific binding. After incubation at 25 °C for 30 min, the samples were resolved by 6% polyacrylamide gel electrophoresis in 0.5× TBE buffer on ice at 100 V for 120 min. The gels were then scanned using a ChemiDoc^TM^ MP imaging system (BioRad, Hercules, CA). To determine the binding of Vp2210 with the *exsC* promoter, we expressed and purified the 6xHis-tagged Vp2210 using the strain pET28a:*vp2210*/BL21 and performed EMSA similarly as VbrR.

### DNase I footprinting assay

DNase I footprinting was performed using purified VbrR protein and *exsC* promoter^71^. The *exsC* promoter was amplified by PCR using primers containing 6-FAM at the 5′ end (EMSA-FAM) (Supplementary Table 2). Two hundred nanograms of the *exsC* promoter from each sample was incubated with different amounts of purified VbrR in a volume of 20 μl. After incubation for 30 min at 25 °C, 0.2 U of DNase I (Promega) and the reaction buffer were added. The mixture was then incubated at 25 °C for 1 min, and 5 μl of DNase I stop solution was added to terminate the reaction. The samples were extracted by phenol/chloroform and then precipitated using ethanol, and the pellets were dissolved in 10 μl of water. Approximately 2 μl of digested DNA was added to 7.9 μl of HiDi formamide (Applied Biosystems, Foster City, CA) and 0.1 μl of GeneScan-500 LIZ size standards (Applied Biosystems). The samples were analyzed using a 3730 DNA Analyzer with a G5 dye set. The results were analyzed using GeneMarker 2.2.0.

### β-galactosidase activity assay

β-galactosidase activity for the strains harboring the construct of P_*exsC*_-lacZ and P_*exsC*Δ50_-lacZ transcription fusion was measured using its substrate o-nitrophenyl-β-D-galactopyranoside (ONPG) (Thermo Fisher Scientific)^71^. The plasmids containing P_*exsC*_-lacZ and P_*exsC*Δ50_-lacZ were transformed to WT, Δ*vbrK*, Δ*vbrK*Δ*vp2210* and Δ*vpa1199-1201*, and the bacteria were cultured in LB with or without nitrite at 37 °C for 4 h under aerobic conditions. The bacterial strains were also incubated on LB agar plates with or without nitrate under anaerobic conditions using an anaerobic box as described above. The pellets were resuspended in 0.7 ml of PM buffer, and 30 μl of chloroform and 30 μl of 0.1% SDS were then added. The solution was vigorously vortexed to lyse the bacterial cells. To initiate the reaction, 200 μl of ONPG (4 mg/ml) was added to the solution. After 10 to 20 min, 0.4 ml of 1 M Na_2_CO_3_ was added to terminate the reaction. The mixture was centrifuged, and the supernatant was used to measure the absorbance at 420 nm. β-galactosidase activity was calculated according to the following formula: A_420_ × 1000 × min^−1^ ×  ml^−1^ × A_600_^−1^.

### Phosphorylation assay

To determine the phosphorylation of VbrK, Δ*vbrK* containing 6xHis-tagged VbrK in a plasmid (Δ*vbrK:*pvbrK*_*6xHis) was grown in LB containing different compounds as described in Fig. 4a. Subsequently, total proteins were isolated by sonication, and phosphorylation was analyzed by measuring the protein mobility shift using a phos-tag gel (FUJIFILM Wako, Richmond, VA)^34^. A regular gel without a phos-tag was used as a control. To determine the phosphorylation of VbrR, Δ*vbrR-*containing 6xHis-tagged VbrR in a plasmid (Δ*vbrR:*p*vbrR_*6xHis) was grown in LB-containing nitrite or nitrate, and a phos-tag gel was used to analyze its phosphorylation. To determine the phosphorylation of VbrK^C86S^, Δ*vbrK-*containing 6xHis-tagged VbrK^C86S^ in a plasmid (Δ*vbrK*:p*vbrK*^C86S^*_*6xHis) was grown in the presence or absence of nitrite, and a phos-tag gel assay was performed similarly.

### In vitro infection

Caco-2 cells were infected with WT, Δ*vbrK*, or ∆*vpa1199-1201* under anaerobic conditions for 4 h in the presence or absence of 1 mg/ml AG (Sigma-Aldrich) or 100 µM 1400 W (Sigma-Aldrich). After infection, the concentration of nitrite and nitrate in the medium was measured by Nitric Oxide Assay Kit according to the manufacturer’s instructions (Thermo Fisher Scientific). Total RNA was isolated from the infected samples, and qRT-PCR was performed to measure the expression of T3SS1 genes. T3SS1 gene expression was also measured for the bacteria in the absence of Caco-2 cells under anaerobic condition. To analyze the expression of proinflammatory cytokines during an in vitro infection, Caco-2 cells were infected with *V. parahaemolyticus* or uninfected under anaerobic conditions for 4 h, and the concentrations of IL-8 and IL-1β in the supernatant were measured by ELISA according to the manufacturer’s instructions (R&D Systems). The cleavage of Caspase-1 during in vitro infection was analyzed by western blotting using anti-caspase-1 antibody (1:200) (Santa Cruz Biotechnology, Dallas, TX). To measure the transcript of IL-8, qRT-PCR was performed for the total RNA isolated after infection as described above. Caco-2 cells were infected with WT or mutant under anaerobic condition for 4 h and LDH assay was performed to measure the cytotoxicity according to the manufacturer’s instruction (Sigma-Aldrich)^21^.

### S-nitrosylation assay

A biotin-switch assay was used to analyze the *S*-nitrosylation of VbrK^72^. Δ*vbrK* harboring a plasmid that expresses 6xHis-tagged VbrK or VbrK^C86S^ was grown in the presence of nitrite or nitrate. The bacterial pellet from 2 ml culture was suspended in 200 µl PBS containing 0.5% Triton X-100 in order to solubilize the proteins. Total proteins from these cultures were obtained after sonication, and free cysteines were then blocked by 20 mM S-methyl methane thiosulfonate (Thermo Fisher Scientific). The samples were then precipitated by acetone and resuspended in HENS buffer (Thermo Fisher Scientific) containing 1% SDS. Biotin-HPDP (Thermo Fisher Scientific) was then added in the presence or absence of sodium ascorbate (Thermo Fisher Scientific), and the samples were then incubated for 2 h at room temperature. Proteins were precipitated with acetone and dissolved in 200 µl PBS, and after the addition of streptavidin beads (Thermo Fisher Scientific), the samples were incubated overnight at 4 °C. Subsequently, the beads were washed, and the proteins were eluted by boiling in loading buffer. Samples prior to streptavidin bead enrichment were used as the input to ensure that equal amounts of proteins were subjected to the enrichment process. SDS-PAGE and immunoblotting were then performed using HRP-conjugated anti-His antibody (1:1000). VbrR *S*-nitrosylation was similarly performed using a Δ*vbrR* harboring plasmid that expresses 6xHis-tagged VbrR. To determine the *S*-nitrosylation of VbrK under anaerobic condition, bacteria were plated on LB agar containing nitrate and placed in the anaerobic box. Bacteria (~0.1 g) were scraped off the plate and resuspended in 200 µl PBS containing 0.5% Triton X-100. Biotin-switch was then performed similarly. *V. parahaemolyticus* strains as described above were grown in LB medium supplemented with nitrite at the concentration of 500 μM. Following 5 hours of growth, a total of 5 ml of culture was centrifuged and the pellet was resuspended in 1 ml of PBS. The bacterial suspension was sonicated and total proteins were loaded onto nickel resin (Thermo Fisher Scientific). After 1 hour of incubation, the nickel resin was washed with PBS three times and then PBS containing 100 mM imidazole was used to obtain the proteins. Subsequently, a biotin-switch assay was performed by adding S-methyl methane thiosulfonate and Biotin-HPDP. For mass spectrometry analysis, proteins obtained from the biotin-switch assay were then digested by trypsin and analyzed by LC–MS/MS. The mass spectrometry data were used to search the VbrK or VbrK^C86S^ protein sequences and biotinylation on cysteine residues.

### In vivo infection assay

The in vivo intestinal colonization assay was performed using an infant rabbit infection model^19,73^. The overnight culture (30 µl) of WT, Δ*vbrK*, Δ*vbrK:*p*vbrK*, and Δ*vbrK:pvbrK*^*C86S*^ was diluted at 1:100 in fresh LB medium and grown to a stationary phase at 30 °C. Approximately 10^9^ CFUs of each strain were intragastrically administered to an infant rabbit. After 18 and 38 h of infection, the CFUs were determined using small intestinal tissues that were homogenized and plated on LB agar. RNA samples were extracted from the small intestine tissues using a RNeasy kit according to the manufacturer’s instruction (Qiagen). The expression of proinflammatory cytokines was measured using the ΔΔCt method^11^. Fluid accumulation was determined after 18 and 38 h of infection by weighing a segment of small intestine before and after the fluid within that segment of small intestine was removed^19^.

### Statistical analysis

For in vitro studies, three independent replications were performed. For animal experiment, five rabbits were included in each group. Data were presented as mean ± standard deviation. Multiple *t* test was used for comparison between two groups. ANOVA was used for comparisons among multiple groups. Multiple *t* test and ANOVA were corrected by the Bonferroni–Dunn method. *P* < 0.05 was considered statistically significant. GraphPad Prism 8 was used for statistical analysis.

### Reporting summary

Further information on research design is available in the Nature Research Reporting Summary linked to this article.

## Supplementary information

Supplementary Information

Reporting Summary

## Data Availability

The genome of *Vibrio parahaemolyticus* RIMD2210633 (https://www.genome.jp/kegg-bin/show_organism?org=vpa) was used as reference for annotation. The RNA-seq data have been deposited in European Nucleotide Archive with accession code PRJEB40026 and PRJEB40744. All relevant data are available from the corresponding author upon request. Source data are provided with this paper.

## Source data

Source Data
