# Supplementary material for: S-nitrosylation-mediated activation of a histidine kinase represses the type 3 secretion system and promotes virulence of an enteric pathogen
Source: Nat Commun. 2020 Nov 13;11:5777. doi: 10.1038/s41467-020-19506-1 (PMC7666205; doi:10.1038/s41467-020-19506-1)
Supplement: Supplementary file 1 — Supplementary Information [file 41467_2020_19506_MOESM1_ESM.pdf]

Supplementary information

S-nitrosylation-mediated activation of a histidine kinase represses the type 3 secretion system and promotes virulence of an enteric pathogen

Dan Gu, Yibei Zhang, Qiyao Wang and Xiaohui Zhou

Contents

Supplementary Fig. 1 Identification of histidine kinase responsible for T3SS1 repression in the LB culture medium

Supplementary Fig. 2 VbrK/VbrR regulates T3SS1 gene expression

Supplementary Fig. 3 EMSA analysis for the binding of VbrR or Vp2210 with promoters

Supplementary Fig. 4 Effect of nitrite and β-lactam on the expression of T3SS1 genes.

Supplementary Fig. 5 KEGG analysis of pathways regulated by nitrite (a) or nitrate (b)

Supplementary Fig. 6 Nitrate affects *exsC* expression under anaerobic condition.

Supplementary Fig. 7 Host-derived nitrite represses T3SS1 gene expression during infection.

Supplementary Fig. 8 Cytotoxicity of indicated *V. parahaemolyticus* strains to Caco-2 cells under anaerobic condition

Supplementary Table 1 Bacterial strains and plasmids used in this study

Supplementary Table 2 Primers used in this study

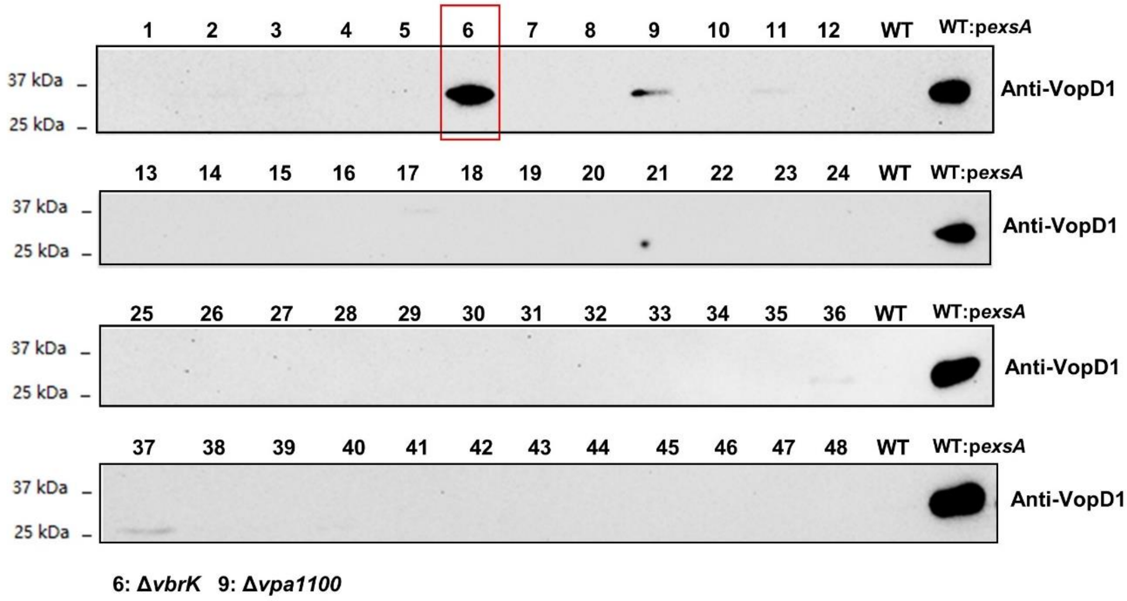

Supplementary Fig. 1 Identification of histidine kinase responsible for T3SS1 repression in the LB culture medium. Genes encoding predicted histidine kinases were deleted individually from the genome of WT *V. parahaemolyticus* and each mutant was grown in LB medium in the presence of carbenicillin (except for Δ*vbrK*) for 3 hours. Culture supernatant was collected and western blot was performed using anti-VopD1 antibody. #1 to #48 on top of the blot indicate mutants lacking *vpa0009, vpa0149, vpa0710, vpa0736, vpa0763, vpa0920 (vbrK), vpa0965, vpa1075, vpa1100, vpa1130, vpa1277, vpa1433, vpa1515, vpa1664, vp0362, vp0487, vp1069, vp1201, vp1212, vp1375, vp1503, vp1712, vp1735, vp1908, vp1968, vp1984, vp2010, vp2478, vp2567, vp2859, vp2874, vpa0020, vpa0182, vpa0826, vpa1196, vpa1731, vpa1220, vpa1229, vpa0675, vp0155, vp0539, vp0570, vp0915, vp1245, vp2252, vp1755, vp1876,* and *vp2229*, respectively. WT:p*exsA* was used as a positive control. Red box indicates the secretion of VopD1 by Δ*vpa0920* (Δ*vbrK*). Representative image of three independent experiments is shown.

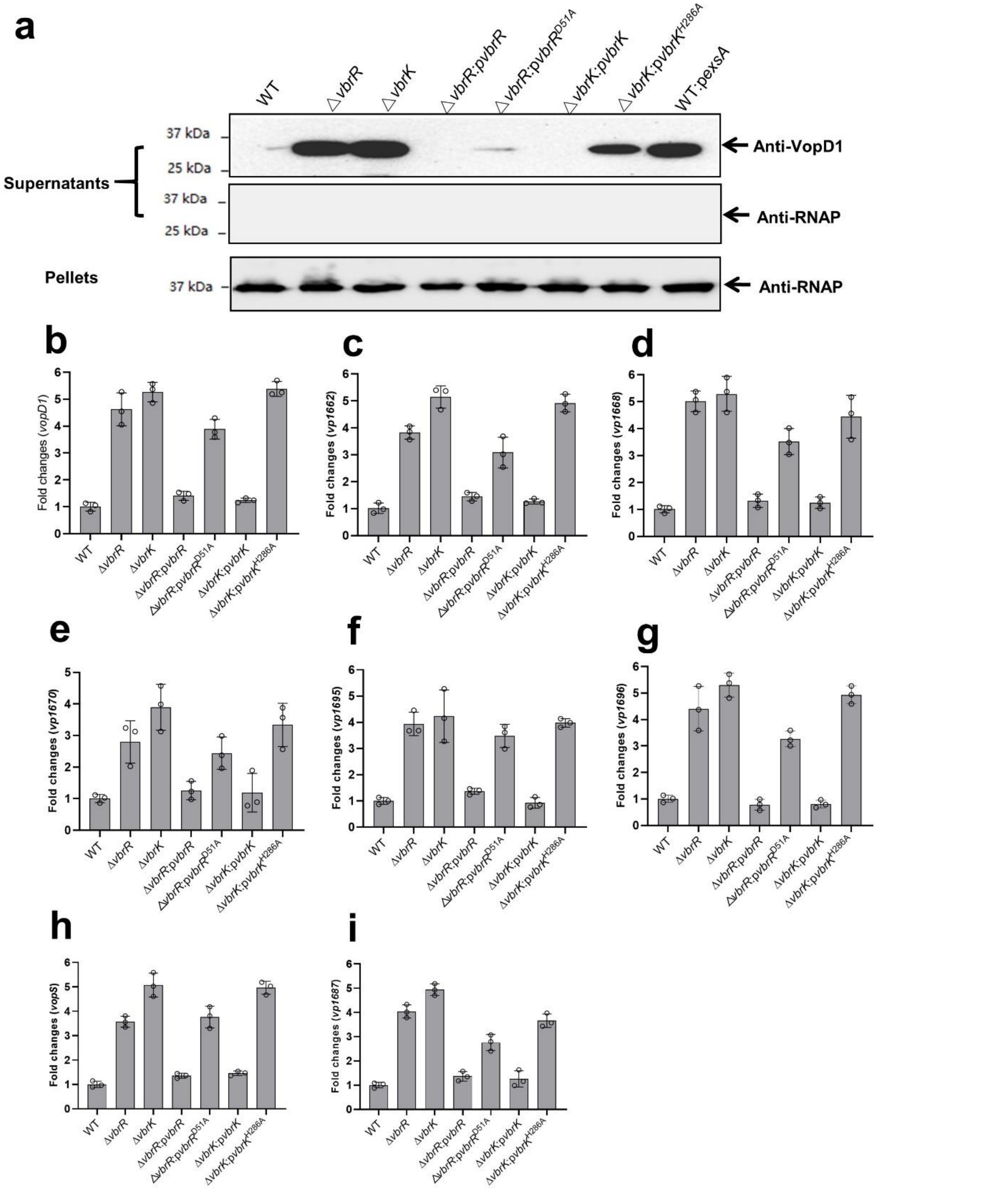

Supplementary Fig. 2 VbrK/VbrR regulates T3SS1 gene expression.

(a) Indicated strains of *V. parahaemolyticus* were grown in the LB medium and supernatant was processed for western blot using anti-VopD1 and anti-RNAP antibodies. Pellet samples were also processed for western blot using anti-RNAP antibody to ensure that the supernatant was prepared from equal amount of bacteria cells across the samples.

(b-i) RNA was isolated from indicated *V. parahaemolyticus* strains cultured in LB medium and qRT-PCR analysis of *vopD1* (b), *vp1662* (c), *vp1668* (d), *vp1670* (e), *vp1695* (f), *vp1696* (g), *vopS* (h) and *vp1687* (i). Bars with standard deviation indicate average fold changes relative to WT. All error bars represent mean ± standard deviation (n=3 biologically independent experiments).

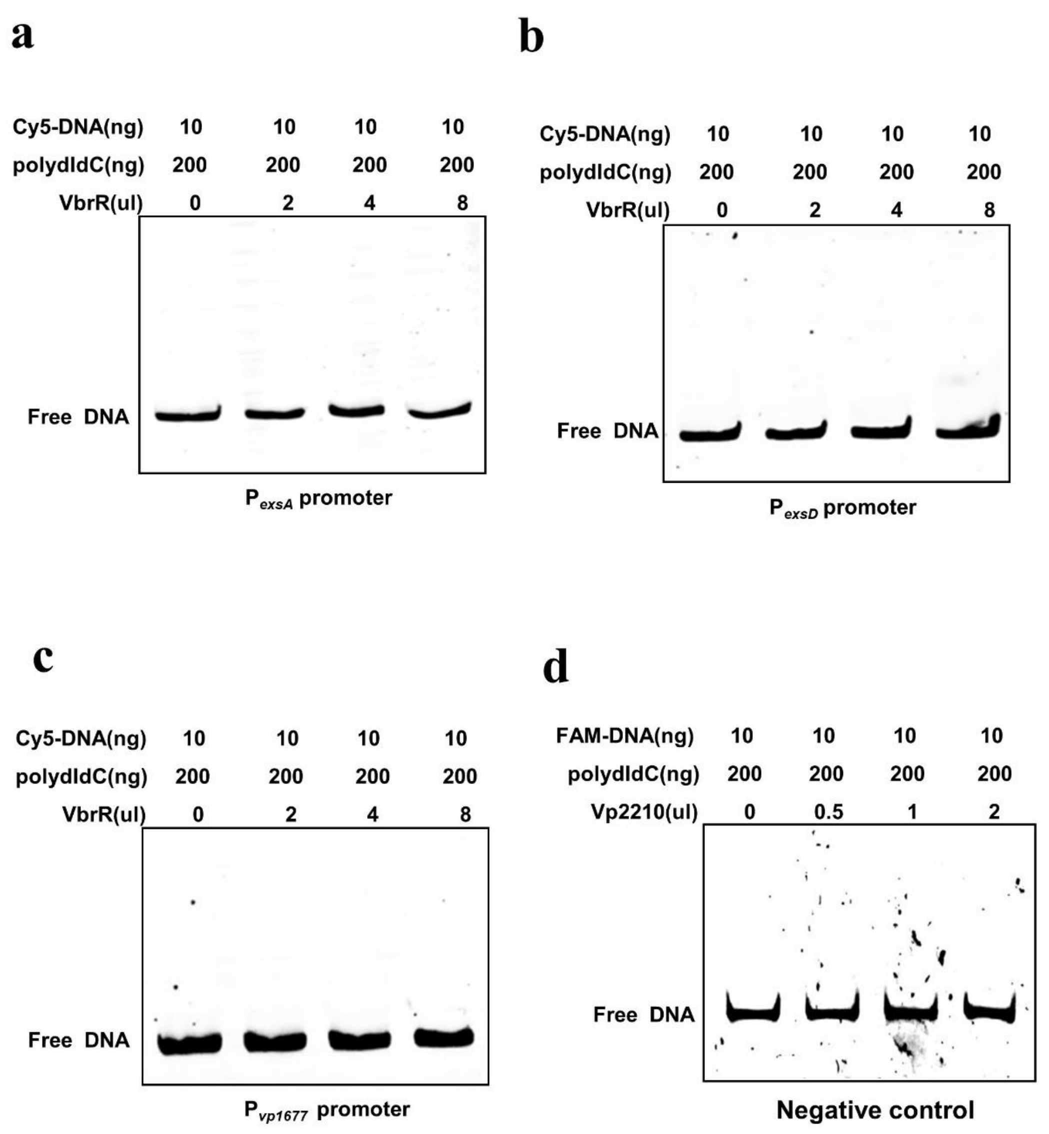

Supplementary Fig. 3 EMSA analysis for the binding of VbrR or Vp2210 with promoters.

(a-c) Purified VbrR protein does not bind promoter of *exsA* (a), *exsD* (b) and *vp1677* (c). (d) Purified Vp2210 protein does not bind negative control promoter of *gyrB*. Representative image of three independent experiments is shown.

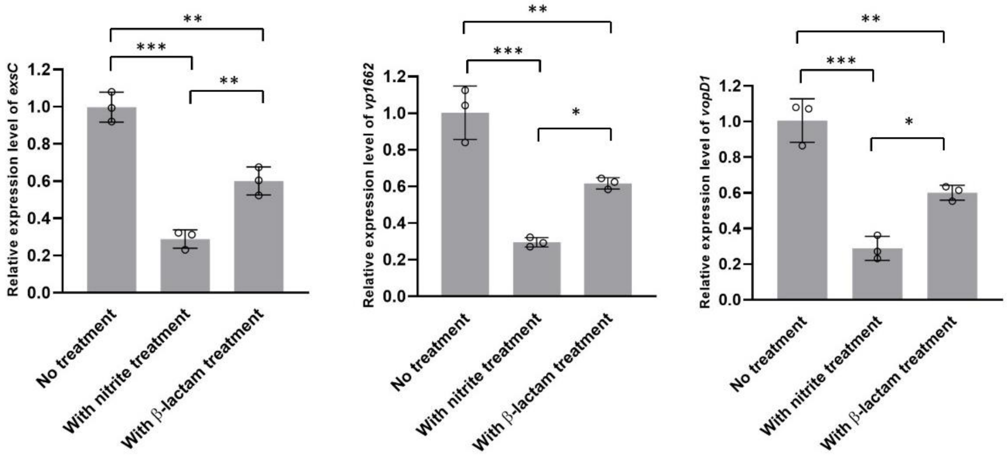

Supplementary Fig. 4 Effect of nitrite and β-lactam on the expression of T3SS1 genes. RNA was isolated from WT *V. parahaemolyticus* cultured in LB medium with or without treatment and qRT-PCR analysis of *exsC* (left panel), *vp1662* (middle panel) and *vopD1* (right panel). Bars with standard deviation (n=3 biologically independent experiments) indicate average fold changes relative to the no treatment condition. Statistical significance was calculated using one-way ANOVA with Bonferroni correction. Asterisks indicate p values *P <0.05, **P <0.005, and ***P< 0.0005.

a

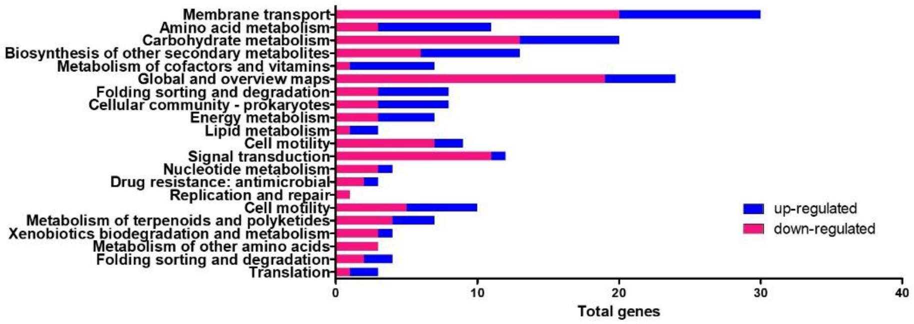

b

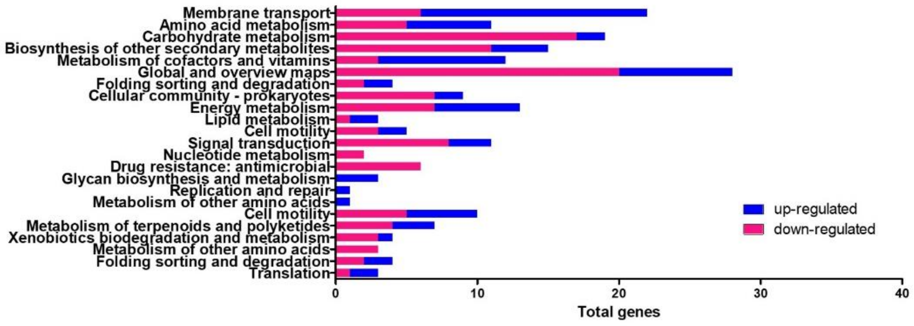

Supplementary Fig. 5 KEGG analysis of pathways regulated by nitrite (a) or nitrate (b). Red color shows the genes that have reduced (by three-fold) transcription level in nitrite- or nitrate-treated samples compared to untreated sample. Blue color shows the genes that have elevated (by three-fold) transcription level in nitrite- or nitrate-treated samples compared to untreated sample.

a

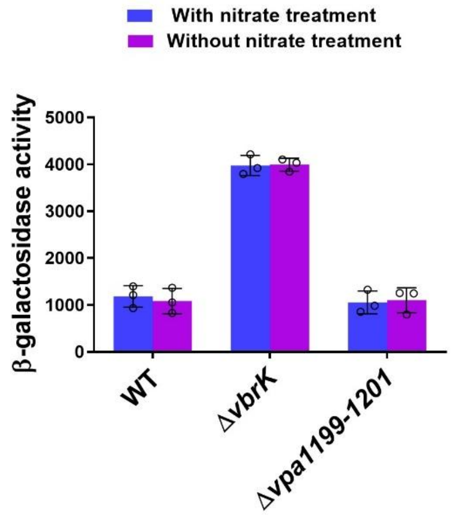

b

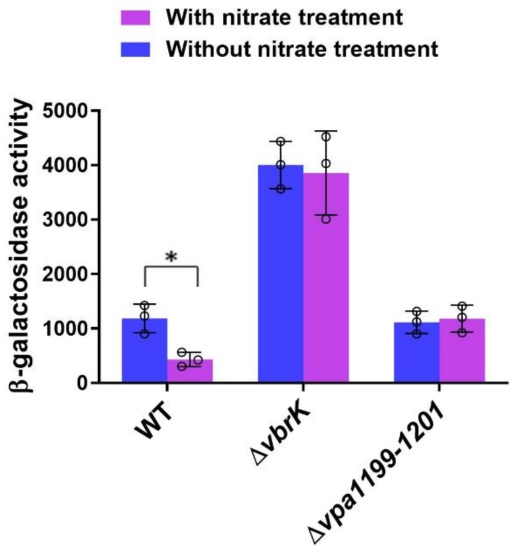

Supplementary Fig. 6 Nitrate affects *exsC* expression under anaerobic condition.

(a) Indicated strains of *V. parahaemolyticus* carrying P_{exsC}-lacZ construct were cultured under aerobic condition in the presence or absence of nitrate. Bars indicate average lacZ activity.

(b) Indicated strains of *V. parahaemolyticus* carrying P_{exsC}-lacZ construct were cultured under anaerobic condition in the presence or absence of nitrate and bars indicate average lacZ activity. (a, b) All error bars represent mean ± standard deviation (n=3 biologically independent experiments). Statistical significance was calculated using two-tailed multiple *t* test with Bonferroni correction. Asterisks indicate p values **P* <0.05, ***P* <0.005, and ****P*< 0.0005.

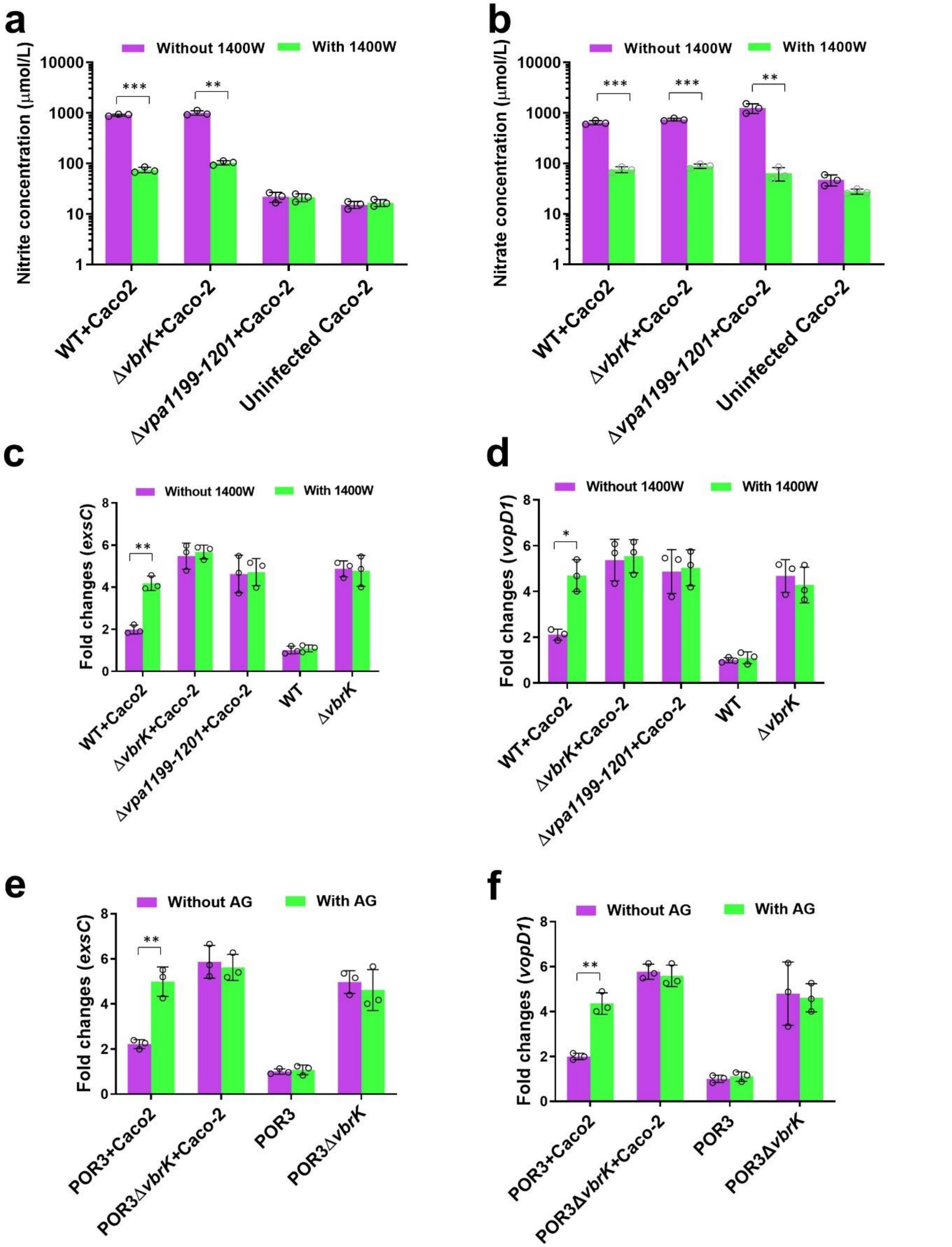
a
Without 1400W
With 1400W
Nitrite concentration (μmol/L)
10000
1000
100
10
1
***
**
WT+Caco2
ΔvbrK+Caco-2
Δvpa1199-1201+Caco-2
Uninfected Caco-2
b
Without 1400W
With 1400W
Nitrate concentration (μmol/L)
10000
1000
100
10
1
***
***
**
WT+Caco2
ΔvbrK+Caco-2
Δvpa1199-1201+Caco-2
Uninfected Caco-2
c
Without 1400W
With 1400W
Fold changes (exsC)
8
6
4
2
0
**
WT+Caco2
ΔvbrK+Caco-2
Δvpa1199-1201+Caco-2
WT
ΔvbrK
d
Without 1400W
With 1400W
Fold changes (vopD1)
8
6
4
2
0
*
WT+Caco2
ΔvbrK+Caco-2
Δvpa1199-1201+Caco-2
WT
ΔvbrK
e
Without AG
With AG
Fold changes (exsC)
8
6
4
2
0
**
POR3+Caco2
POR3ΔvbrK+Caco-2
POR3
POR3ΔvbrK
f
Without AG
With AG
Fold changes (vopD1)
8
6
4
2
0
**
POR3+Caco2
POR3ΔvbrK+Caco-2
POR3
POR3ΔvbrK

Supplementary Fig. 7 Host-derived nitrite represses T3SS1 gene expression during infection.

(a) Nitrite concentration measured in the culture medium of Caco-2 cells infected with the indicated strains in the presence or absence of 1400W. All error bars represent mean ± standard deviation (n=3 biologically independent experiments).

(b) Nitrate concentration measured in the culture medium of Caco-2 cells infected with the indicated strains in the presence or absence of 1400W. All error bars represent mean ± standard deviation (n=3 biologically independent experiments).

(c, d) Quantitative RT-PCR analysis of T3SS1 gene expression in *V. parahaemolyticus* during infection. Caco-2 cells were infected with WT (WT+Caco-2), Δ*vbrK* (Δ*vbrK*+Caco-2) or Δ*vpa1199-1201* (Δ*vpa1199-1201*+Caco-2) and RNA was collected from the infected samples for qRT-PCR analysis of *exsC* (C) and *vopD1* (D). Transcripts of these two genes in WT and Δ*vbrK* in the LB medium were also included as controls. Bars indicate average fold changes relative to WT under LB growth condition without 1400W treatment. All error bars represent mean ± standard deviation (n=3 biologically independent experiments).

(e, f) Caco-2 cells were infected with POR3 (POR3+Caco-2) and POR3Δ*vbrK* (POR3Δ*vbrK*+Caco-2) and RNA was collected from the infected samples for qRT-PCR analysis of *exsC* (E) and *vopD1* (F). Bars indicate average fold changes relative to POR under LB growth condition without 1400W treatment. All error bars represent mean ± standard deviation (n=3 biologically independent experiments).

(a-f) Statistical significance was calculated using two-tailed multiple *t* test with Bonferroni correction. Asterisks indicate p values *P <0.05, **P <0.005, and ***P< 0.0005.

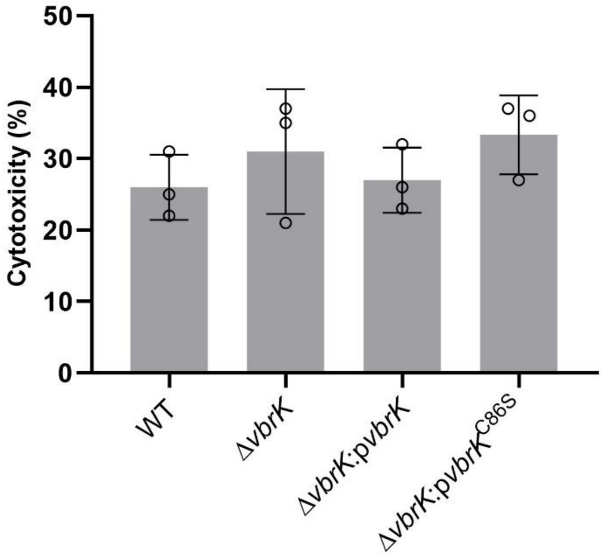

Supplementary Fig. 8. Cytotoxicity of indicated *V. parahaemolyticus* strains to Caco-2 cells under anaerobic condition. Caco-2 cells were infected for 4 hours and LDH assay was performed to measure the cytotoxicity. All error bars represent mean ± standard deviation (n=3 biologically independent experiments).

Supplementary Table 1. Bacterial strains and plasmids used in this study

Strain or plasmid	Relevant characteristics	Reference
E. coli		
DH5α λ*pir*	For plasmid propagation	Laboratory collection
SM10 λ*pir*	For plasmid conjugation	Laboratory collection
BL21(DE3)	Host strain for protein expression	Laboratory collection
pET28a:*vp2210*/BL21	BL21 carrying pET28a that expresses *vp2210*	This study
V. parahaemolyticus		
RIMD 2210633	WT	Laboratory collection
POR3	WT with deletion of T3SS2 and TDH	Laboratory collection
WT:p*exsA*	WT expressing the *exsA* gene	Laboratory collection
Δ*vbrK*	WT with in-frame deletion of *vbrK*	[34]
Δ*vbrR*	WT with in-frame deletion of *vbrR*	[34]
Δ*vbrR*/Δ*vp2210*	WT with in-frame deletion of *vbrR* and *vp2210*	This study
Δ*vpa1199-1201*	WT with in-frame deletion of *vpa1199*, *vpa1200*, and *vpa1201*	This study
Δ*vbrK:pvbrK_6xHis*	Δ*vbrK* harboring pMMB207 that expresses *vbrK*_6xHis gene	[34]
Δ*vbrR:pvbrR_6xHis*	Δ*vbrR* harboring pMMB207 that expresses *vbrR*_6xHis	[34]
Δ*vbrK*:p*vbrK*^{H286A}*_6xHis*	Δ*vbrK* harboring pMMB207 that expresses *vbrK*^{H286A}_6xHis	[34]
Δ*vbrR*:P*vbrR*^{D51A}*_6xHis*	Δ*vbrR* harboring pMMB207 that expresses *vbrR*^{D51A}_6xHis	[34]
Δ*vbrK*:P*vbrK*^{C86S}*_6xHis*	Δ*vbrK* harboring pMMB207 that expresses *vbrK*^{C86S}_6xHis	This study
Δ*vbrK*Δ*exsA*	WT with in-frame deletion in *vbrK* and *exsA*	This study
Δ*vbrK*Δ*exsC*	WT with in-frame deletion in *vbrK* and *exsC*	This study
Δ*vbrK:pexsD*	Δ*vbrK* harboring pMMB207 that expresses *exsD*_6xHis gene	This study
Δ*vbrR:pexsD*	Δ*vbrR* harboring pMMB207 that expresses *exsD*_6xHis gene	This study
WT/*exsC_6xHis*	6xHis tag inserted at the C-terminus of *exsC* in the genome of WT	This study
Δ*vbrR*/*exsC_6xHis*	6xHis tag inserted at the C-terminus of *exsC* in the genome of Δ*vbrR*	This study

Δ*vbrK/exsC_6xHis*	6xHis tag inserted at the C-terminus of *exsC* in the genome of Δ*vbrK*	This study
VP0055-his/WT	WT harboring pMMB207 that expresses *vp0055*-6xHis gene	This study
VP0404-his/WT	WT harboring pMMB207 that expresses *vp0404*-6xHis gene	This study
VP2210-his/WT	WT harboring pMMB207 that expresses *vp2210*-6xHis gene	This study
VP2232-his/WT	WT harboring pMMB207 that expresses *vp2232*-6xHis gene	This study
VP-2358-his/WT	WT harboring pMMB207 that expresses *vp2358*-6xHis gene	This study
VP2553-his/WT	WT harboring pMMB207 that expresses *vp2553*-6xHis gene	This study
VP2578-his/WT	WT harboring pMMB207 that expresses *vp2578*-6xHis gene	This study
VP2670-his/WT	WT harboring pMMB207 that expresses *vp2670*-6xHis gene	This study
VP2953-his/WT	WT harboring pMMB207 that expresses *vp2953*-6xHis gene	This study
VPA1690-his/WT	WT harboring pMMB207 that expresses *vpa1690*-6xHis gene	This study
P_{exsC}-lacZ/WT	WT harboring pDM8 that contains the fusion of WT *exsC* promoter with promoterless *lacZ* gene	This study
P_{exsC}-lacZ/Δ*vbrR*	Δ*vbrR* harboring pDM8 that contains the fusion of WT *exsC* promoter with promoterless *lacZ* gene	This study
P_{exsC}-lacZ/Δ*vbrK*	Δ*vbrK* harboring pDM8 that contains the fusion of WT *exsC* promoter with promoterless *lacZ* gene	This study
$P_{exsC\Delta 50}$-lacZ/WT	WT harboring pDM8 that contains the fusion of mutant *exsC* promoter (exsCΔ50) with promoterless *lacZ* gene	This study
$P_{exsC\Delta 50}$-lacZ/Δ*vbrR*	Δ*vbrR* harboring pDM8 that contains the fusion of mutant *exsC* promoter (exsCΔ50) with promoterless *lacZ* gene	This study
$P_{exsC\Delta 50}$-lacZ/Δ*vbrK*	Δ*vbrK* harboring pDM8 that contains the fusion of mutant *exsC* promoter (exsCΔ50) with promoterless *lacZ* gene	This study
P_{exsC}-lacZ/Δ*vbrK*/Δ*vp2210*	Δ*vbrK*/Δ*vp2210* harboring pDM8 that contains the fusion of WT *exsC* promoter with promoterless *lacZ* gene	This study
P_{exsC}-lacZ/Δ*vpa1199-1201*	Δ*vpa1199-1201* harboring pDM8 that contains the fusion of WT *exsC* promoter with promoterless *lacZ* gene	
**Plasmids**		
pDM4	Suicide vector (*pir* dependent, R6K, *SacBR*) for making gene deletion mutant	Laboratory collection
pMMB207	Plasmid for complementation of the mutant	Laboratory collection
pDM8	Plasmid with promoterless *lacZ* gene	Laboratory collection

Supplementary Table 2 Primers used in this study

Primer name	Primer sequence (5' to 3')	Function
VPA1199-1201-UP-F:	GCTTATCGATACCGTCGACCCTCGATAGAACTGGGAAAAGTAGCACAACC	For deletion of vpa1199-1201
VPA1199-1201-UP-R	TTTGTCTCCTCACAAACGCACGTCTTGTCATTTTC	For deletion of vpa1199-1201
VPA1199-1201-DOWN-F	TGCGTTTGTGAGGAGACAAACCATGAGTACT	For deletion of vpa1199-1201
VPA1199-1201-DOWN-R	CACTAGTGACGCGTACTCGAGCAATGCAGAACACAAAGAAGG	For deletion of vpa1199-1201
EMSA-FAM	TGCCTGCAGGTCGACGAT	For EMSA
*exsA*EMSA-F	TGCCTGCAGGTCGACGATACTACTCATTCAGAAGCCTG	For EMSA
*exsA*EMSA-R	TTTCTACCCTTCATAATTTT	For EMSA
*exsC*EMSA-F	TGCCTGCAGGTCGACGATACGTTCCACGTACAACACGT	For EMSA
*exsC*EMSA-R	AGAAACAGTCCTTTTGAGAA	For EMSA
exsD-EMSA-F	TGCCTGCAGGTCGACGATACTCAAAGCTATCGACGTCG	For EMSA
exsD-EMSA-R	CCCTTTCGAACACTCCAGAA	For EMSA
vp1677-EMSA-F	TGCCTGCAGGTCGACGATTGTAAAAAATATGCGCAATG	For EMSA
vp1677-EMSA-R	TCACTTGCACTCCTTTCGTA	For EMSA
*gyrB*EMSA-F	TGCCTGCAGGTCGACGATGCGAAAGAAGTGGGTGCAAG	For EMSA
*gyrB*EMSA-R	TTTGATGTACGCGCCCTCTT	For EMSA
ExsC-his1	GCTTATCGATACCGTCGACCCTCGACACGATATCGAATATGTTTCCCGAT	For insertion of 6xHis to the C-terminus of *exsC* in the genome
ExsC-his2	AAGACACCTAGTGATGATGATGATGATGAACTCTCAGATCTAAACTTTGAGGC	For insertion of 6xHis to the C-terminus of *exsC* in the genome
ExsC-his3	TCATCATCACTAGGTGTCTTATGTCTAATGACATC	For insertion of 6xHis to the C-terminus of *exsC* in the genome
ExsC-his4	CACTAGTGACGCGTACTCGATGCTGGATCACTACCAGAATACTAA	For insertion of 6xHis to the C-terminus of *exsC* in the genome
VP1662-RT-F	CACCAAAGCCAGCACTAGGA	For RT-PCR
VP1662-RT-R	TGTTTCCCACGAAGACTCGG	For RT-PCR
VP1668-RT-F	TCTTGGCGTTCGCTCCATAG	For RT-PCR
VP1668-RT-R	CGCCAATCAGAGCAAGAACG	For RT-PCR
VP1670-RT-F	GAACTCGCGGCTGCTAAAAC	For RT-PCR

VP1670-RT-R	CAGGATGAGAGGCGAGTGAC	For RT-PCR
VP1695-RT-F	CAGTGCGGTTCTTTTCCAGC	For RT-PCR
VP1695-RT-R	GTGAATCGGGCATCGAGCTA	For RT-PCR
VP1696-RT-F	CCTAGGTACGGAATGTCGCC	For RT-PCR
VP1696-RT-R	GATCCCAGATGGTGGTGTGG	For RT-PCR
VP1687-RT-F	GCCAACAAAACCGACTTCCTC	For RT-PCR
VP1687-RT-R	TGATTGACCAAGACACGGCA	For RT-PCR
VP1686 (VopS)-RT-F	AAACTCGCAACGTTGGCTTC	For RT-PCR
VP1686 (VopS)-RT-R	ACTACCGCGAAAGTGCTGAA	For RT-PCR
VP1656 (VopD1)-RT-F	CTGAGCTTGTCCCTGTTCGT	For RT-PCR
VP1656 (VopD1)-RT-R	GGCAAACTCAGCATTGGTGG	For RT-PCR
gyrB-RT-F	TGACAGCCGTTGTTTCGGTA	For RT-PCR
gyrB-RT-R	AGTCTGCAAGTTTGCCTGGT	For RT-PCR
exsCEMSA23-F	TGCCTGCAGGTCGACGATTTAACATAGTATTTAATTTTTAAGA	For EMSA
exsCEMSA48-F	TGCCTGCAGGTCGACGATTAACACAATTCTAATTCTTCATCGC	For EMSA
exsCEMSA73-F	TGCCTGCAGGTCGACGATTTGTCGATATAAACCAAAATCCCAC	For EMSA
exsCEMSA98-F	TGCCTGCAGGTCGACGATCATAATAGAATTTAATAACCGTTAA	For EMSA
exsCEMSA123-F	TGCCTGCAGGTCGACGATTCTTTATATGCACCAGCACTCATTT	For EMSA
exsCEMSA148-F	TGCCTGCAGGTCGACGATGTAAGATTAGAAGCAAGGAGTCAAT	For EMSA
exsCEMSA173-F	TGCCTGCAGGTCGACGATGGTTTCCGGCCAAAATATAA	For EMSA
exsCEMSA198-F	TGCCTGCAGGTCGACGATATAACTAACAACCAAAATTG	For EMSA
exsCEMSA165-F	TGCCTGCAGGTCGACGATGAGTCAATTCTGGGTTTCCG	For EMSA
exsCEMSA111-R	TAAATTCTATTATGGTGGGA	For EMSA
Vp0055-his-F	AGCTCGGTACCCGGGGATCCTCTAGTAAGGAGGTAGGATAATAATGGC AACGACTTCAGCCCTA	For vp0055 overexpression
Vp0055-his-R	TCTCATCCGCCAAAACAGCCAAGCTTTAGTGATGATGATGATGATGTGC GTGTGCCATTCTTGATTG	For vp0055 overexpression
VP0404-his-F	AGCTCGGTACCCGGGGATCCTCTAGTAAGGAGGTAGGATAATAATGGAT CAAAATCCGCAGTCAC	For vp0404 overexpression
VP0404-his-R	TCTCATCCGCCAAAACAGCCAAGCTTTAGTGATGATGATGATGATGTTCG TCGAGGAAGCTGCGCAG	For vp0404 overexpression
VP2210-his-F	AGCTCGGTACCCGGGGATCCTCTAGTAAGGAGGTAGGATAATAATGGAG GCTCCTGTGGCCATA	For vp2210 overexpression

VP2210-his-R	TCTCATCCGCCAAAACAGCCAAGCTTTAGTGATGATGATGATGATGTCCA TTTTTTTGTCCTCT	For vp2210 overexpression
VP2232-his-F	AGCTCGGTACCCGGGGATCCTCTAGTAAGGAGGTAGGATAATAGTGAATA AAGCGATAACCTATG	For vp2232 overexpression
VP2232-his-R	TCTCATCCGCCAAAACAGCCAAGCTTTAGTGATGATGATGATGATGGTCAT TTTGTGTCCACGC	For vp2232 overexpression
VP2358-his-F	AGCTCGGTACCCGGGGATCCTCTAGTAAGGAGGTAGGATAATAATGATGAG TGATAGCCCACAA	For vp2358 overexpression
VP2358-his-R	TCTCATCCGCCAAAACAGCCAAGCTTTAGTGATGATGATGATGATGTAGTTG CTCCGTATGCATTG	For vp2358 overexpression
VP2553-his-F	AGCTCGGTACCCGGGGATCCTCTAGTAAGGAGGTAGGATAATAATGAGTATC AGCAACACAGTA	For vp2553 overexpression
VP2553-his-R	TCTCATCCGCCAAAACAGCCAAGCTTTAGTGATGATGATGATGATGGGATTT CACGAAGTCGACG	For vp2553 overexpression
VP2578-his-F	AGCTCGGTACCCGGGGATCCTCTAGTAAGGAGGTAGGATAATAATGAACGAG CAGCTGACCGATC	For vp2578 overexpression
VP2578-his-R	TCTCATCCGCCAAAACAGCCAAGCTTTAGTGATGATGATGATGATGGCGCTGT AAAAGAGGTTTG	For vp2578 overexpression
VP2670-his-F	AGCTCGGTACCCGGGGATCCTCTAGTAAGGAGGTAGGATAATAATGAAACCTT CATTACAACTC	For vp2670 overexpression
VP2670-his-R	TCTCATCCGCCAAAACAGCCAAGCTTTAGTGATGATGATGATGATGAAGTAGG CGTTTACGCTG	For vp2670 overexpression
VP2953-his-F	AGCTCGGTACCCGGGGATCCTCTAGTAAGGAGGTAGGATAATAATGACAAAAG AAGCGTATCCG	For vp2953 overexpression
VP2953-his-R	TCTCATCCGCCAAAACAGCCAAGCTTTAGTGATGATGATGATGATGGAACTCAC CAACGGCCAGT	For vp2953 overexpression
VPA1690-his-F	AGCTCGGTACCCGGGGATCCTCTAGTAAGGAGGTAGGATAATAATGGAAAAACA AAAACGTTACG	For vpa1690 overexpression
VPA1690-his-R	TCTCATCCGCCAAAACAGCCAAGCTTTAGTGATGATGATGATGATGATGGTTTCC TTGCTGCGCG	For vpa1690 overexpression
PexsC-lacZ-F	ATTGCTGCAGGTCGACGGATCCGGGGAATTATTCTACAGATGGACAGCAATAACG	For construction *exsC* promoter-LacZ fusion
PexsC-lacZ-R	GTCGACCTGCAGCCCAAGCTTATCGATTCGAGAAACAGTCCTTTTGAGAATTTTA	For construction *exsC* promoter-LacZ fusion

PexsCΔ50-lacZ-2	ATTCTATTATGATCTTAAAAATTAAATACTATG	For construction *exsC*Δ50 promoter-LacZ fusion
PexsCΔ50-lacZ-3	ATTTTTAAGATCATAATAGAATTTAATAACCGT	For construction *exsC*Δ50 promoter-LacZ fusion
