# Supplementary material for: S-nitrosylation-mediated activation of a histidine kinase represses the type 3 secretion system and promotes virulence of an enteric pathogen
Source: Nat Commun. 2020 Nov 13;11:5777. doi: 10.1038/s41467-020-19506-1 (PMC7666205; doi:10.1038/s41467-020-19506-1)
Supplement: Supplementary file 2 — Reporting Summary [file 41467_2020_19506_MOESM2_ESM.pdf]

nature research

Corresponding author(s): Xiaohui Zhou

Last updated by author(s): Oct 4, 2020

Reporting Summary

Nature Research wishes to improve the reproducibility of the work that we publish. This form provides structure for consistency and transparency in reporting. For further information on Nature Research policies, see our Editorial Policies and the Editorial Policy Checklist.

Statistics

For all statistical analyses, confirm that the following items are present in the figure legend, table legend, main text, or Methods section.

n/a	Confirmed	
☐	☒	The exact sample size (*n*) for each experimental group/condition, given as a discrete number and unit of measurement
☐	☒	A statement on whether measurements were taken from distinct samples or whether the same sample was measured repeatedly
☐	☒	The statistical test(s) used AND whether they are one- or two-sided<br>*Only common tests should be described solely by name; describe more complex techniques in the Methods section.*
☒	☐	A description of all covariates tested
☐	☒	A description of any assumptions or corrections, such as tests of normality and adjustment for multiple comparisons
☐	☒	A full description of the statistical parameters including central tendency (e.g. means) or other basic estimates (e.g. regression coefficient) AND variation (e.g. standard deviation) or associated estimates of uncertainty (e.g. confidence intervals)
☐	☒	For null hypothesis testing, the test statistic (e.g. *F*, *t*, *r*) with confidence intervals, effect sizes, degrees of freedom and *P* value noted<br>*Give P values as exact values whenever suitable.*
☒	☐	For Bayesian analysis, information on the choice of priors and Markov chain Monte Carlo settings
☒	☐	For hierarchical and complex designs, identification of the appropriate level for tests and full reporting of outcomes
☒	☐	Estimates of effect sizes (e.g. Cohen's *d*, Pearson's *r*), indicating how they were calculated

Our web collection on statistics for biologists contains articles on many of the points above.

Software and code

Policy information about availability of computer code

Data collection	No software was used
Data analysis	GraphPad Prism 8 was used to draw graphs and analyze statistical data. RNA-seq data was analyzed by Rockhopper program 2.0.3 and edgeR 3.30.3. Genemarker 2.2.0 was used to analyze footprinting results.

For manuscripts utilizing custom algorithms or software that are central to the research but not yet described in published literature, software must be made available to editors and reviewers. We strongly encourage code deposition in a community repository (e.g. GitHub). See the Nature Research guidelines for submitting code & software for further information.

Data

Policy information about availability of data

All manuscripts must include a data availability statement. This statement should provide the following information, where applicable:
- Accession codes, unique identifiers, or web links for publicly available datasets
- A list of figures that have associated raw data
- A description of any restrictions on data availability

Genome of Vibrio parahaemolyticus RIMD2210633 was retrieved from the following website: https://www.genome.jp/kegg-bin/show_organism?org=vpa.
All data supporting the findings of this study are available within the paper, its supplementary information files, and source data are provided as a Source Data file linked to this article. Source data underlying Figs. 1(BCDEF), 2(ABDF), 3(ABCD), 4(ABCDEF), 6(ABCDEF), 7(ABCFGHI), 8(ABCDEF), 9(ABCD) and Supplementary Figs. 1, 2(ABCDEFGHI), 3(ABCD), 4, 6(AB),7(ABCDEF), and 8 are provided as Source data file. Source data are provided with this paper. All relevant data can be requested from the corresponding author upon reasonable request.

Field-specific reporting

Please select the one below that is the best fit for your research. If you are not sure, read the appropriate sections before making your selection.

[x] Life sciences [ ] Behavioural & social sciences [ ] Ecological, evolutionary & environmental sciences

For a reference copy of the document with all sections, see nature.com/documents/nr-reporting-summary-flat.pdf

Life sciences study design

All studies must disclose on these points even when the disclosure is negative.

Sample size	Sample sizes for each experiment were stated in Statistical analysis. Sample sizes were determined empirically, and similar in size to most existing studies in the same field. For in vitro assays, n=3. For in vivo rabbit experiments, n = 5 of rabbits were used for each group (Deng, H., Yang, W., Zhou, Z. et al. Nat Commun 11, 4951 (2020)). No statistical method was used to predetermine sample size.
Data exclusions	No data was excluded.
Replication	Every experiment at replication was successful. All experiments were performed for a minimum of three independent replicates with similar results. For in vivo experiment, reproducibility was verified by similar results of bacterial CFU in the small intestine and fluid accumulation for individual animal within the group. All replications were successful.
Randomization	Rabbits were randomly groups.
Blinding	Because all data was quantification of CFU and relative expression, and are not subjective, blinding was not relevant. All experiments were performed by the same researcher for data consistency, blinding was not possible.

Reporting for specific materials, systems and methods

We require information from authors about some types of materials, experimental systems and methods used in many studies. Here, indicate whether each material, system or method listed is relevant to your study. If you are not sure if a list item applies to your research, read the appropriate section before selecting a response.

Materials & experimental systems

n/a	Involved in the study	
[ ]	[x]	Antibodies
[ ]	[x]	Eukaryotic cell lines
[x]	[ ]	Palaeontology and archaeology
[ ]	[x]	Animals and other organisms
[x]	[ ]	Human research participants
[x]	[ ]	Clinical data
[x]	[ ]	Dual use research of concern

Methods

n/a	Involved in the study	
[x]	[ ]	ChIP-seq
[x]	[ ]	Flow cytometry
[x]	[ ]	MRI-based neuroimaging

Antibodies

Antibodies used	Mouse monoclonal Anti-RNA polymerase (BioLegend, 663104), Anti-mouse IgG (Thermo Fisher Scientific, 31430), HRP-conjugated anti-His antibody (R&D System, MAB050H), Caspase-1 antibody (Santa Cruz Biotechnology, sc-56036), Mouse polyclonal anti-VopD1 antibody was described in previous studies (Zhou et al. Virulence, 2010, 1: 260-72. PMID: 21178451, DOI: 10.4161/viru.1.4.12318).
Validation	All antibodies were validated according to respective manufacturer's information. The quality test data was shown on the manufacturers' websites:<br>Mouse monoclonal Anti-RNA polymerase (BioLegend, 663104)<br>https://www.biolegend.com/en-us/products/purified-anti-e-coli-rna-polymerase-alpha-antibody-14680<br>Anti-mouse IgG (Thermo Fisher Scientific, 31430)<br>https://www.thermofisher.com/antibody/product/Goat-anti-Mouse-IgG-H-L-Secondary-Antibody-Polyclonal/31430<br>HRP-conjugated anti-His antibody (R&D System, MAB050H)<br>https://www.rndsystems.com/products/his-tag-horseradish-peroxidase-conjugated-antibody-ad1110_mab050h<br>Caspase-1 antibody (Santa Cruz Biotechnology, sc-56036)<br>https://www.scbt.com/p/caspase-1-antibody-14f468<br>Mouse polyclonal anti-VopD1 antibody<br>Mouse polyclonal anti-VopD1 antibody was described in previous studies (Zhou et al. Virulence, 2010, 1: 260-72. PMID: 21178451, DOI: 10.4161/viru.1.4.12318)<br>https://www.tandfonline.com/doi/full/10.4161/viru.1.4.12318

Eukaryotic cell lines

Policy information about cell lines

Cell line source(s)	Caco-2 cells were obtained from ATCC (HTB-37™).
Authentication	Caco-2 cells were not authenticated.
Mycoplasma contamination	Caco-2 cell line was negative for Mycoplasma contamination.
Commonly misidentified lines (See ICLAC register)	N/A

Animals and other organisms

Policy information about studies involving animals; ARRIVE guidelines recommended for reporting animal research

Laboratory animals	Infant rabbits Strain NewZealand, Age: 1-2 days. Sex: male or female (random)
Wild animals	The study did not involve wild animals.
Field-collected samples	The study did not involve filed-collected samples.
Ethics oversight	Animal experiments were approved by the Institutional Animal Care and Use Committee (IACUC) of University of Connecticut (Protocol #A13-060).

Note that full information on the approval of the study protocol must also be provided in the manuscript.
